# An Integrated Analysis of Transcriptomics and Metabolomics Elucidates the Role and Mechanism of TRPV4 in Blunt Cardiac Injury

**DOI:** 10.3390/metabo15080512

**Published:** 2025-07-31

**Authors:** Liancong Gao, Liu Han, Xiangyu Ma, Huiyan Wang, Mutan Li, Jianhui Cai

**Affiliations:** 1 Clinical Medical College, Jilin Medical University, Jilin 132013, China; gaolc@jlmu.edu.cn (L.G.);; 2 College of Pharmacy, Jilin Medical University, Jilin 132013, China; hanliu@jlmu.edu.cn (L.H.);; 3 Jilin Provincial Technology Collaborative Innovation Center of Antibody Engineering, Jilin Medical University, Jilin 132013, China

**Keywords:** blunt cardiac injury, TRPV4, transcriptomics, metabolomics, mechanism

## Abstract

Background/Objectives: Blunt cardiac injury (BCI) is a severe medical condition that may arise as a result of various traumas, including motor vehicle accidents and falls. The main objective of this study was to explore the role and underlying mechanisms of the TRPV4 gene in BCI. Elucidating the function of TRPV4 in BCI may reveal potential novel therapeutic targets for the treatment of this condition. Methods: Rats in each group, including the SD control group (SDCON), the SD blunt-trauma group (SDBT), the TRPV4 gene-knockout control group (KOCON), and the TRPV4 gene-knockout blunt-trauma group (KOBT), were all freely dropped from a fixed height with a weight of 200 g and struck in the left chest with a certain energy, causing BCI. After the experiment, the levels of serum IL-6 and IL-1β were detected to evaluate the inflammatory response. The myocardial tissue structure was observed by HE staining. In addition, cardiac transcriptome analysis was conducted to identify differentially expressed genes, and metabolomics studies were carried out using UHPLC-Q-TOF/MS technology to analyze metabolites. The results of transcriptomics and metabolomics were verified by qRT-PCR and Western blot analysis. Results: Compared with the SDCON group, the levels of serum IL-6 and IL-1β in the SDBT group were significantly increased (*p* < 0.001), while the levels of serum IL-6 and IL-1β in the KOBT group were significantly decreased (*p* < 0.001), indicating that the deletion of the TRPV4 gene alleviated the inflammation induced by BCI. HE staining showed that myocardial tissue injury was severe in the SDBT group, while myocardial tissue structure abnormalities were mild in the KOBT group. Transcriptome analysis revealed that there were 1045 upregulated genes and 643 downregulated genes in the KOBT group. These genes were enriched in pathways related to inflammation, apoptosis, and tissue repair, such as p53, apoptosis, AMPK, PPAR, and other signaling pathways. Metabolomics studies have found that TRPV4 regulates nucleotide metabolism, amino-acid metabolism, biotin metabolism, arginine and proline metabolism, pentose phosphate pathway, fructose and mannose metabolism, etc., in myocardial tissue. The combined analysis of metabolic and transcriptional data reveals that tryptophan metabolism and the protein digestion and absorption pathway may be the key mechanisms. The qRT-PCR results corroborated the expression of key genes identified in the transcriptome sequencing, while Western blot analysis validated the protein expression levels of pivotal regulators within the p53 and AMPK signaling pathways. Conclusions: Overall, the deletion of the TRPV4 gene effectively alleviates cardiac injury by reducing inflammation and tissue damage. These findings suggest that TRPV4 may become a new therapeutic target for BCI, providing new insights for future therapeutic strategies.

## 1. Introduction

With the rapid development of science and technology, blunt cardiac injuries (BCIs) caused by motor vehicle accidents, falls from heights, and impacts from blunt instruments are increasing. BCI is one of the main causes of death and disability from trauma. Therefore, research on the impact and mechanism of blunt injuries on the heart is of great significance and has attracted widespread attention from all sectors of society [1].

The American Heart Association/European Resuscitation Committee (AHA/ERC) states that BCI-induced myocardial damage or arrhythmia can culminate in high mortality rates even in the absence of preexisting structural heart disease, a paradox likely explained by unrecognized severe functional impairment or delayed complications [2]. Bhi Cakchi et al. found that myofibrillar loss occurred in cardiomyocytes in the cardiac contusion group, and serum cardiac troponin I (C-TI) levels were measured by ELISA. Compared with the control group, apoptosis, Tumor Necrosis Factor-alpha (TNF-α), Creatine Kinase (CK), Creatine Kinase-MB (CK-MB), Lactate Dehydrogenase (LDH), and C-TI levels all increased in the cardiac contusion group [3]. Lae-Young J et al. reported a marked decrease in heart rate and blood pressure in a male patient with chest trauma. Echocardiography conducted two hours post-injury demonstrated an ejection fraction of 53%, along with hypokinesia observed in the basal septal segment, mid-to-basal posterolateral wall, and anterolateral wall. These findings are indicative of regional myocardial dysfunction. Myocardial enzyme levels were significantly elevated, while coronary angiography demonstrated no significant lesions [4]. Nevertheless, these studies predominantly focused on macro-level detection and did not delve into the underlying mechanisms of the injury.

Transient receptor potential vanilloid 4 (TRPV4) is a non-selective cation-permeable channel, primarily facilitating the influx of calcium ions (Ca^2+^). This channel plays a critical role in intracellular Ca^2+^ signaling within cardiac cells and can be activated by various stimuli, including mechanical stress, small-molecule drugs, and hypotonic solutions [5]. Research has indicated that TRPV4 contributes to multiple pathological processes by influencing mitochondrial function in cardiomyocytes and vascular barrier integrity. For example, studies by Sebastien Chaigne et al. have demonstrated that this channel is involved in cardiac fibrosis, myocardial hypertrophy, heart failure, myocardial infarction, and arrhythmia, suggesting its potential as a therapeutic target for cardiovascular diseases [6]. Regarding the role of TRPV4 in cardiac fibrosis, investigations by RK. Adapala et al. have revealed that TRPV4 mediates fibrosis through regulating cardiac fibroblast differentiation and Rho/MRTF-A pathway-mediated collagen synthesis. Further evidence shows that genetic ablation of TRPV4 suppresses abnormal fibroblast activation after myocardial infarction via the above mechanisms, thereby mitigating adverse cardiac remodeling [7]. However, although TRPV4′s involvement in cardiovascular diseases is well-documented, the mechanisms underlying TRPV4-mediated passive cardiac injury remain incompletely understood.

Omics analysis techniques are increasingly utilized to identify potential biomarkers and elucidate the causes and mechanisms underlying diseases [8]. Metabolomics enables the analysis of small-molecule metabolites, thereby reflecting the biological metabolic characteristics associated with disease states, and is extensively applied in biomarker discovery [9]. Metabolomics encompasses the systematic measurement of numerous metabolites—including nutrients, drugs, signaling mediators, and their metabolites—in blood, urine, tissue extracts, or other bodily fluids. Transcriptomics focuses on the transcription of all genes within cells, encompassing mRNA and non-coding RNA. Through high-throughput sequencing technology, it provides comprehensive and rapid gene expression information, thus revealing gene expression regulation [10]. Transcriptomic studies can investigate gene function and structure at multiple levels, uncovering the molecular mechanisms of specific biological processes and diseases. The integration of metabolomics and transcriptomics has emerged as a critical approach for studying complex biological processes, enabling a more comprehensive analysis of biomolecular functions and regulatory mechanisms, and aiding in the elucidation of disease pathogenesis and identification of potential therapeutic targets [11]. Thus, transcriptomics and metabolomics can enhance the diagnosis of BCI by characterizing dynamic molecular profiles, including specific differential gene expression and distinct metabolite markers that reflect underlying pathophysiological processes. However, the mechanistic connections between these profiles and injury identification necessitate further investigation.

The aim of this study was to investigate the role and influence of TRPV4 in a rat model of BCI. Additionally, through an integrated transcriptomic and metabolomics analysis, we sought to elucidate the mechanisms underlying the effects of the TRPV4 channel on BCI, thereby providing insights for the prevention and treatment of such injuries.

## 2. Materials and Methods

### 2.1. Animals and Experiment Design

SD wild-type (WT) rats (6–8 weeks of age) and SD-background TRPV4-knockout rats (TRPV4^−/−^) were included from the Antibody Experimental Center of Jilin Medical College. TRPV4-knockout rats were obtained using CRISPR/CAS9 technology. All the experimental rats were female rats. There were 12 female SD wild-type (WT) rats, weighing 200 g ~ 250 g; there were 12 female TRPV4-knockout (TRPV4-/-) rats, 6 ~ 8 weeks old, weighing 200 g ~ 250 g. The rats were randomly divided into the following 4 groups: SDCON (*n* = 6), SDBT (*n* = 6), KOCON (*n* = 6), and KOBT (*n* = 6). This experiment was approved by the Animal Experiment Ethics Committee (Ethics Number: 2020-KJT005) and carried out at the Antibody Experimental Center of Jilin University of Medicine. The model of cardiac contusion caused by BCI in rats was established according to reference [3]. Under ether anesthesia, a 200 g cylindrical object (balance weights) was dropped onto the chest of a rat from a height of 1.0 m. Consequently, 1.96 J of energy was transferred to the right hemithorax [E = mgh (g = 9.8 m/s^2^); m = mass of the cylinder (0.2 kg), g = gravitational acceleration (9.8 m/s^2^), h = drop height (1.0 m)].

### 2.2. Serum and Tissue Collection

After 24 h of BCI, under deep anesthesia (ether), the abdominal aorta was exposed via a midline laparotomy. Blood samples (1–2 mL) were collected using a 22-gauge needle inserted into the abdominal aorta, avoiding vessel laceration to minimize blood loss and tissue damage. After the heart was removed, part of it was fixed with 4% paraformaldehyde, and the other part was immediately stored in a refrigerator at −80 °C for subsequent metabolomic, transcriptomic, and Western blot experimental analyses.

The blood was then allowed to clot at room temperature for 1 h, followed by centrifugation using a high-speed refrigerated centrifuge (4 °C, 3000 r/min, 15 min) to separate the serum. Separated serum samples were stored at −20 °C. Thereafter, the hearts of the rats in each group were rapidly excised and placed in liquid nitrogen for preservation until further analysis. The levels of inflammatory factors, including IL-1β and IL-6, in the serum were measured using an ELISA kit.

The heart tissue was fixed in 4% paraformaldehyde for 24 h, dehydrated with a graded ethanol series, and subsequently embedded in paraffin wax. Sections of 4 μm thickness were obtained, and the pathological section machine was purchased from Shanghai Leica Instruments Co., Ltd. (Shanghai, China) (RM2016). The tissue sections were stained with hematoxylin and eosin, and myocardial histology was analyzed using optical microscopy.

### 2.3. Extraction and Isolation of Total RNA

Total RNA was extracted from heart tissue using TRIzol^®^ reagent (Invitrogen, Carlsbad, CA, USA) in accordance with the manufacturer’s protocol. Subsequently, the RNA concentration and integrity were assessed using the Illumina TruSeq™ RNA Sample Preparation Kit (Illumina, San Diego, CA, USA) [12].

### 2.4. Transcriptome Measurements and Analysis

The RNA-seq library was constructed and sequenced on the Illumina NovaSeq 6000 platform provided by Majorbio Bio-Pharm Technology Co., Ltd. (Shanghai, China). Raw data quality control was performed using FASTP software (version 0.19.5) to remove low-quality sequences and adapter contamination, thereby generating high-quality clean reads. These clean reads were subsequently aligned to the reference genome using HISAT2 (version 2.1.0). The reference genome was obtained from the Ensembl genome database (http://asia.ensembl.org/Rattus_norvegicus/Info/Index (accessed on 22 January 2024)). Gene expression quantification was conducted using the Transcripts Per Million (TPM) normalization method. Significant differential gene expression was identified based on stringent criteria: adjusted *p*-value (padj) < 0.05 and |log2 fold change| > 1. The biological interpretation of these differentially expressed genes was facilitated through functional annotation workflows implemented on the Majorbio Cloud Platform (https://cloud.majorbio.com/), an integrated analytical environment that provides open access to Gene Ontology (GO) and Kyoto Encyclopedia of Genes and Genomes (KEGG) pathway resources.

### 2.5. Untargeted LC-MS Metabolomics Analysis

Myocardial tissue samples (80 mg per group) were processed by homogenizing with 200 μL of ultra-pure water for one minute. Subsequently, 800 μL of a methanol–acetonitrile solution (1:1, *v*/*v*) was added to the mixture, which underwent two cycles of cryogenic sonication, each lasting 30 min. Protein precipitation was facilitated by incubating the mixture at −20 °C for one hour, followed by centrifugation at 12,000× *g* for 20 min at a temperature of 4 °C. The resulting supernatant was collected and lyophilized to obtain metabolite extracts, which were stored at −80 °C until further analytical procedures were conducted.

The metabolites present in each lyophilized specimen were analyzed using an Ultra-High-Performance Liquid Chromatography coupled with Quadrupole Time-of-Flight mass spectrometry (UHPLC-Q-TOF/MS) platform. Separation was accomplished with an Agilent Technologies 1290 Infinity UHPLC system equipped with a hydrophobic interaction column, operating at a temperature of 25 °C, and employing a mobile phase flow rate of 0.3 mL/min. The aqueous mobile phase comprised 25 mmol/L ammonium acetate and 25 mmol/L ammonium hydroxide dissolved in ultra-pure water, while the organic phase consisted solely of acetonitrile. Electrospray ionization (ESI) enabled dual-polarity (positive/negative mode) spectral acquisition. Compound characterization involved dual verification through high-precision mass accuracy (<25 ppm error margin) and alignment of MS/MS spectral patterns with reference databases, including the Human Metabolome Database (HMDB, http://www.hmdb.ca), METLIN (https://metlin-nl.scripps.edu/), and a proprietary database developed by Majorbio (https://v.majorbio.com/).

Before the initiation of analytical procedures, datasets underwent Pareto scaling normalization. The subsequent discriminant analysis utilized orthogonal partial least squares discriminant analysis (OPLS-DA) via R software (v3.3.1), a methodology that effectively segregates systematic variation into predictive and orthogonal components. This analytical approach enhances model interpretability and predictive capacity by eliminating noise variables that are independent of classification. The identification of differential metabolites was based on variable importance in projection (VIP) scores derived from OPLS-DA models, combined with established statistical thresholds (VIP > 1 and *p* < 0.05). The Metabo Analyst 4.0 platform facilitated further processing, implementing t-tests for the identified metabolites and conducting metabolic pathway enrichment analyses through integration with the KEGG database and Majorbio Cloud Platform. Pathways exhibiting *p*-values below 0.05 were considered significantly affected by TRPV4 deficiency, indicating potential biological relevance.

### 2.6. Real-Time Quantitative PCR (RT-qPCR)

Total RNA was isolated following the protocol outlined in Section 2.3. The quality of the RNA was assessed for integrity by evaluating its purity and concentration using a μDrop Plate (Thermo Scientific, Juensuu, Finland) prior to cDNA synthesis. Amplification reactions were performed with SYBR Green Master Mix (ThermoFisher), utilizing 1 μL of synthesized cDNA and GAPDH as the endogenous control. The thermal cycling parameters included an initial denaturation phase at 95 °C for 60 s, followed by 40 cycles consisting of a 15 s denaturation at 95 °C, a 30 s annealing at 55 °C, and a 30 s extension at 60 °C. Target gene expression levels were quantified through 2^−ΔΔCt^ analysis with normalization against the GAPDH reference values. The primer sequences employed are detailed in Supplementary Data Table S1.

### 2.7. Protocol for Western Blot Analysis

The protein concentration was determined after the lysis of the cardiac tissue samples was completed. After determining the protein concentration, gel electrophoresis was performed with a sample loading mass of 20 µg. After electrophoresis electroporation, the polyvinylidene fluoride (PVDF) membranes were sealed with milk and treated with anti-AMPK (1:1000) (Affinity Biosciences, Changzhou, China) and p-AMPK (1:1000) (Affinity Biosciences, Changzhou, China). The primary antibodies of p53 (1:400) (Proteintech Group, Inc., Wuhan, China) and β-actin (1:500) (Proteintech Group, Inc., Wuhan, China) were incubated overnight at 4 °C. After washing the TBST membrane, the secondary antibody was incubated and developed with Enhanced Chemiluminescence (ECL) luminescent solution.

### 2.8. Statistical Analysis

Experimental results are presented as mean values ± standard deviations obtained from six independent trials. Statistical comparisons were conducted using GraphPad Prism 9.5.0 (GraphPad Software Inc., San Diego, CA, USA) to evaluate the significance of differences between experimental conditions and their corresponding control groups. All multi-group comparisons were analyzed using one-way ANOVA (and nonparametric or mixed). A probability threshold of *p* < 0.05 was established as the criterion for identifying statistically significant variations.

## 3. Results

### 3.1. Analysis of Serum Biochemical Parameters and Heart Histopathology

**Figure 1:**
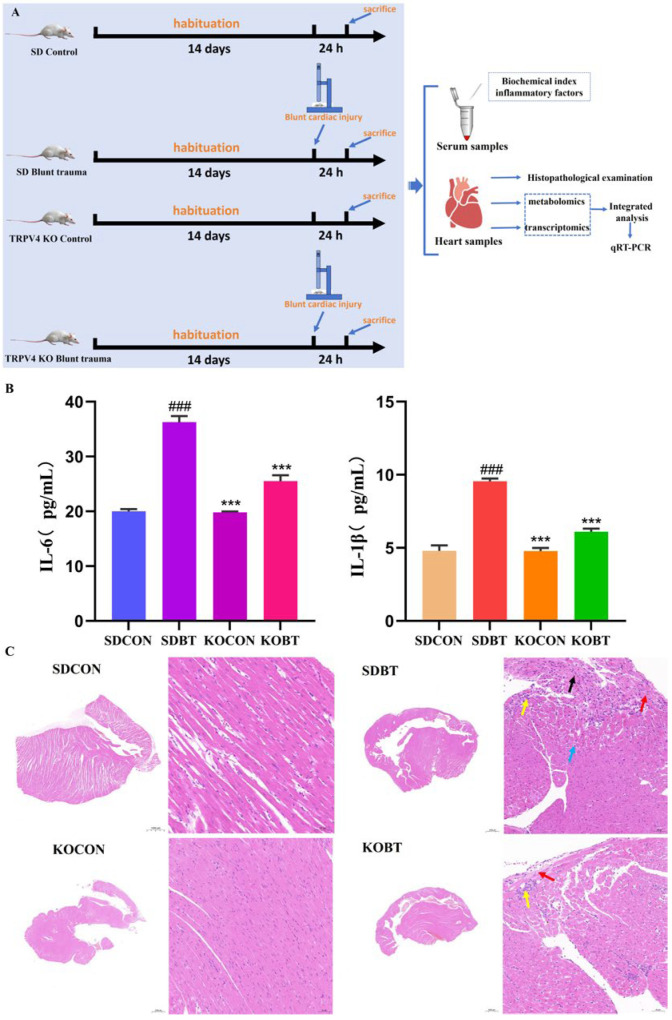
Flowchart of the experimental protocol, serum indices, and histopathological analysis of the heart: (**A**) flowchart of rat experiments; (**B**) serum inflammatory factors IL-6 and IL-1β levels; (**C**) HE-stained cardiac tissue at 200× magnification. The black arrow indicates atrophy of myocardial cells, the blue arrow indicates mild vacuolar degeneration of the cells, the yellow arrow indicates inflammatory cell infiltration, the red arrow indicates fibrous tissue hyperplasia. (### or ***) *p* < 0.001. # Compared with the SDCON group. * compared with the SDBT group.

Figure 1A illustrates the overall experimental design and procedure. Figure 1B presents the detection results of serum IL-6 and IL-1β levels in each group. The findings indicate that the serum concentrations of IL-6 and IL-1β were significantly elevated in the SDBT group compared to the SDCON group (*p* < 0.001). Furthermore, the serum levels of IL-6 and IL-1β in the KOCON group were markedly lower than those in the SDBT group (*p* < 0.001), suggesting that TRPV4 gene deletion effectively mitigates the inflammatory response induced by BCI. Histopathological analysis via HE staining (Figure 1C) revealed that the myocardial tissue structure in the SDCON group was largely intact, with neatly and tightly arranged myofibers and no evident signs of degeneration or inflammatory cell infiltration. Conversely, the SDBT group exhibited abnormal myocardial tissue architecture, characterized by disordered cardiomyocyte arrangement in certain regions, extensive cellular atrophy (indicated by black arrows), minimal vacuolar degeneration (indicated by blue arrows), inflammatory cell infiltration (indicated by yellow arrows), and fibrous tissue hyperplasia (indicated by red arrows). In the KOCON group, the myocardial tissue structure remained normal, with orderly and compactly arranged cardiomyocytes and no apparent degeneration, inflammatory cell infiltration, or fibrosis. In the KOBT group, the myocardium displayed only mild structural abnormalities, including loosely and irregularly arranged cardiomyocytes in some areas, multifocal interstitial inflammatory cell infiltration (indicated by yellow arrows), and minor fibrous tissue hyperplasia in the epicardium (indicated by red arrows). Compared to the SDBT group, the extent of myocardial tissue damage was substantially reduced in the KOBT group.

### 3.2. Analysis of Gene Expression in Myocardial Tissue

#### 3.2.1. Quality Control of RNA-Seq Reads

Transcripts from the SDCON, the SDBT, and the KOBT group were analyzed via RNA-seq. This study investigated the accumulation patterns of mRNA in the cDNA library and evaluated their potential biological significance. In summary, the processed datasets demonstrated substantial sequencing depth exceeding 6.14 Gb per sample, with base call accuracy metrics surpassing 93.9% for the Q30 scores, as detailed in Supplementary Table S2. Following rigorous quality assessment, high-confidence reads underwent genomic alignment verification, achieving mapping efficiencies between 96.85% and 97.68% across all biological replicates relative to the reference genome. Complete alignment metrics are documented in Supplementary Table S3, confirming data robustness for subsequent analytical workflows.

#### 3.2.2. Differentially Expressed Genes (DEGs) of Myocardial Tissues

**Figure 2:**
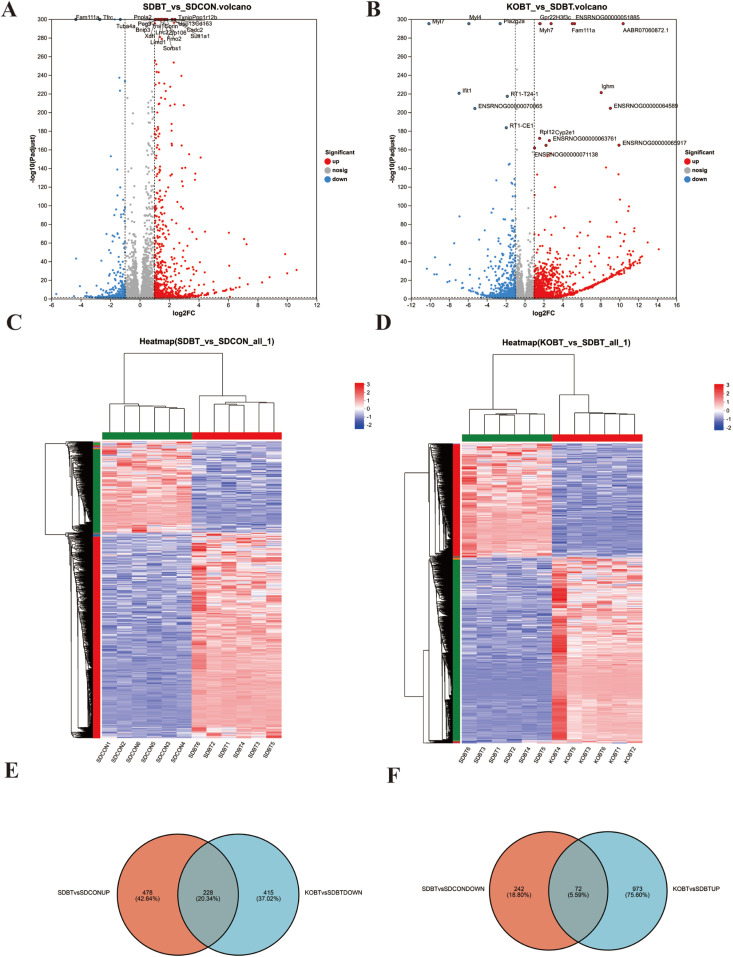
The screening results of DEGs in rat heart tissue samples from the SDCON, SDBT, and KOBT groups: (**A**) volcano plot of DEGs of SDBT vs. SDCON; (**B**) volcano plot of DEGs of KOBT vs. SDBT; (**C**) cluster heatmap of DEGs of SDBT vs. SDCON; (**D**) cluster heatmap of DEGs of KOBT vs. SDBT; (**E**) Venn diagram of DEGs between (SDBT vs. SDCON up) and (KOBT vs. SDBT down); (**F**) Venn diagram revealing common downregulated DEGs (SDBT vs. SDCON) intersecting with upregulated DEGs (KOBT vs. SDBT).

Figure 2A displays the differentially expressed genes (DEGs) observed between the SDBT and SDCON experimental groups. Comparative evaluation demonstrated 706 transcripts exhibiting marked upregulation and 304 showing substantial downregulation in SDBT specimens relative to SDCON controls. Subsequently, a comparative analysis of gene expression differences was conducted between the KOBT and SDBT groups. The findings revealed that, compared with the SDBT group, 1045 genes were significantly upregulated and 643 genes were significantly downregulated in the KOBT group (Figure 2B). Hierarchical clustering patterns for the differentially expressed genes across both comparative analyses are illustrated in Figure 2C,D. The relationships among experimental groups were further clarified through Venn diagram representations (Figure 2E,F), which identified a total of 228 coregulated transcripts that overlap between the upregulated SDBT/SDCON genes and downregulated KOBT/SDBT genes, as well as 72 inversely correlated genes common to the downregulated SDBT/SDCON and upregulated KOBT/SDBT datasets. These molecular signatures were subsequently subjected to comprehensive functional characterization via Gene Ontology annotation and KEGG pathway mapping analyses.

#### 3.2.3. Key Regulatory Role of TRPV4 at the Transcriptional Level

**Figure 3:**
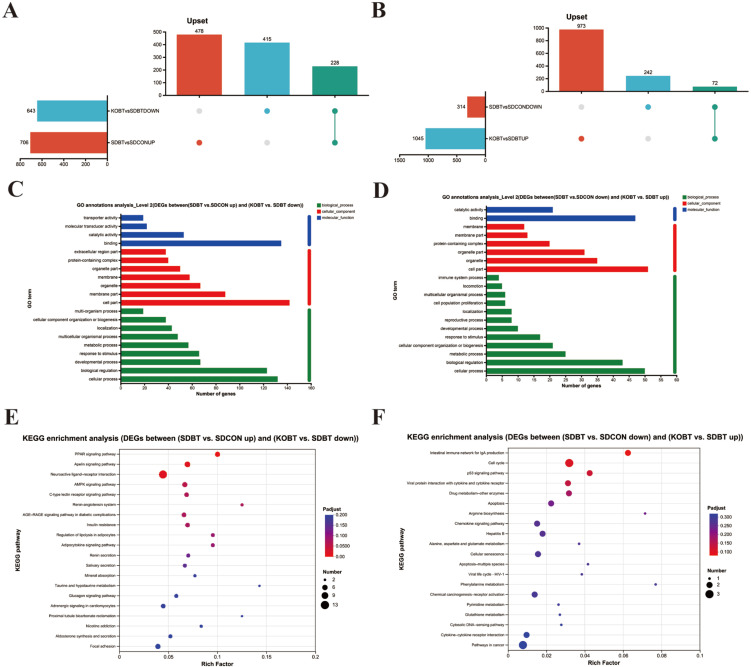
Overall analysis of DEGs: (**A**) upset plot of DEGs downregulated by KOBT vs. SDBT and upregulated by SDBT vs. SDCON; (**B**) upset plot of DEGs upregulated by KOBT vs. SDBT and downregulated by SDBT vs. SDCON; (**C**) GO functional annotation characterization of SDBT-activated/KOBT-repressed DEGs; (**D**) GO functional annotation analysis of SDBT-inhibited/KOBT-induced DEGs; (**E**) KEGG enrichment analysis of SDBT-stimulated/KOBT-suppressed DEGs; (**F**) KEGG enrichment analysis of SDBT-depressed/KOBT-activated DEGs.

To elucidate the primary regulatory mechanisms of KOBT on SDBT-induced cardiac damage, we conducted a comparative analysis of two distinct gene expression clusters. The first cluster comprised transcripts that exhibited upregulated expression during SDBT exposure but were downregulated following KOBT genetic modification. In contrast, the second cluster included genes that displayed opposite expression patterns. As shown in Figure 3A, 228 transcripts demonstrated reduced expression in SDBT-exposed samples but were markedly upregulated upon KOBT administration. Conversely, Figure 3B indicates that 72 genetic markers showed elevated activity after SDBT treatment but were significantly suppressed following KOBT application.

GO functional annotation analysis was performed on genes that were inhibited by SDBT but activated by KOBT (Figure 3C). The results revealed significant enrichment in the molecular function (MF) categories of “binding”, “catalytic activity”, and “molecular function regulator”. Cell component (CC) types were predominantly concentrated in “cell part”, “membrane part”, and “organelle”. The classifications of biological processes (BP) were significantly linked to “cellular processes”, “biological regulation”, and “developmental processes”.

The GO functional annotation analysis of genes upregulated by SDBT but downregulated under KOBT treatment (Figure 3D) revealed significant enrichment in the MF classifications, particularly in “molecular binding” and “enzyme catalytic activity”. CC classifications indicated predominant localization to “cellular structures”, “subcellular organelles”, and “organellar components”. BP classifications showed pronounced clustering in “cellular regulatory mechanisms”, “homeostatic control processes”, and “biochemical transformation pathways”. Comprehensive GO annotation datasets have been systematically compiled and are provided in Supplementary Tables S4 and S5.

Subsequently, the KEGG pathway enrichment analysis uncovered distinct regulatory patterns between the treatment groups. Genes that were upregulated during SDBT exposure but downregulated after KOBT genetic modification showed significant enrichment in inflammation- and apoptosis-related pathways, including PPAR signaling, AMPK signaling, Apelin signaling, neuroactive ligand–receptor interaction, and C-type lectin receptor signaling (Figure 3E). This regulatory reversal implies the potential therapeutic mechanism of KOBT in mitigating SDBT-induced myocardial inflammation and programmed cell death. Conversely, differentially expressed genes (DEGs) suppressed by SDBT yet activated by KOBT intervention demonstrated enrichment in immunoglobulin A (IgA)-related biological processes, particularly those involving cell cycle regulation, p53 signaling cascades, apoptotic mechanisms, and the intestinal immune network for IgA production (Figure 3F). The bidirectional transcriptional modifications observed in the comparative transcriptome profiles indicate pathway-specific reversal effects of KOBT treatment on SDBT-induced molecular alterations. These differential pathway activations appear to be fundamentally linked to TRPV4′s functional involvement in BCI pathology, suggesting potential therapeutic targets within these signaling networks.

### 3.3. Analysis of Accumulated Metabolites in Cardiac Tissue

#### 3.3.1. Systematic Identification and Functional Annotation of Metabolites

**Figure 4:**
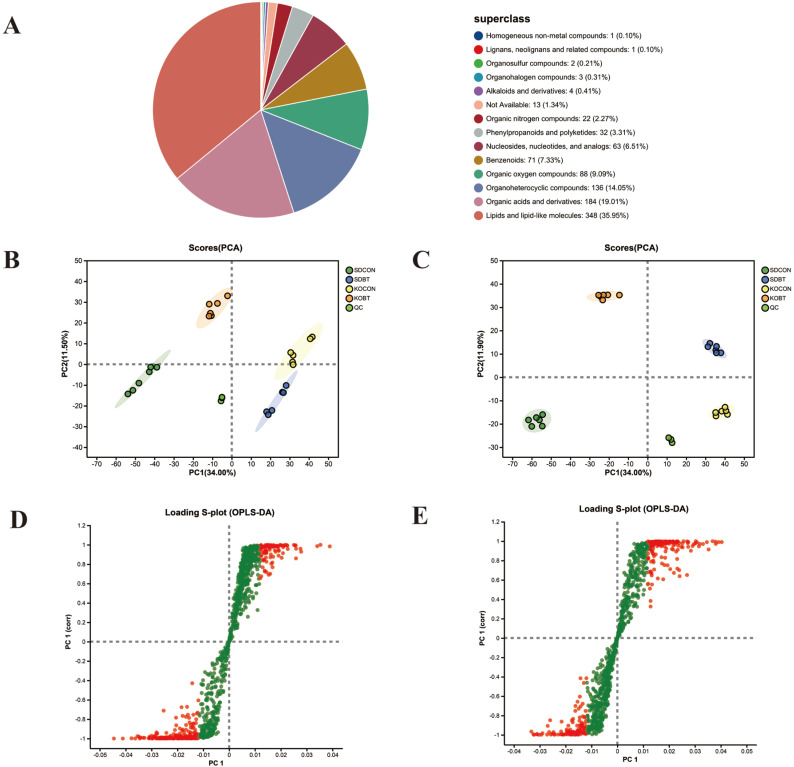
Overall comprehensive characterization of DAMs: (**A**) HMDB-based classification of metabolite categories; (**B**) PCA analysis of positive-ion-mode metabolites; (**C**) PCA analysis of negative-ion-mode metabolites; (**D**) OPLS-DA analysis comparing SDBT and SDCON groups; (**E**) OPLS-DA evaluation between KOBT and SDBT groups.

UHPLC-Q-TOF/MS analysis detected 531 metabolites in positive-ion mode and 507 in negative-ion mode, as documented in Table S6. Subsequently, these metabolites were functionally annotated using a comprehensive database. Specifically, the HMDB provided annotation information for 982 metabolites. Based on the classification results, the major categories include lipids and their derivatives (35.95%), organic acids and their derivatives (19.01%), organic heterocyclic compounds (14.05%), and organic oxygen compounds (9.09%) (as depicted in Figure 4A).

#### 3.3.2. Differential Accumulation of Metabolites (DAMs) in Cardiac Tissue

To examine comprehensive changes in cardiometabolic dynamics among various cohorts, we performed multivariate analyses on the metabolic datasets. In the principal component analysis (PCA) score plot, significant differences were observed in the cardiometabolic profiles of the experimental groups (as depicted in Figure 4B,C). Additionally, in the OPLS-DA score plots obtained in both positive- and negative-ion modes, distinct separations were evident between the SDBT group and the SDCON group, as well as between the KOBT group and the SDBT group (Supplementary Figures S1 and S2), suggesting KOBT genetic modification effectively mitigates myocardial impairment. Similarly, the S-map of OPLS-DA revealed significant differences in cardiometabolic characteristics between the SDBT group and the SDCON group, as well as between the KOBT group and the SDBT group. Based on these results, VIP values for metabolites were calculated (Figure 4D,E).

To clarify the biochemical pathways linked to the observed differential metabolites (DAMs), we conducted an enrichment analysis of metabolic pathways through the KEGG database. The analysis demonstrated that metabolic pathways such as “carbohydrate digestion and absorption,” “fatty acid degradation,” and “amino sugar and nucleotide sugar metabolism” were significantly enriched when comparing differentially expressed metabolites (DEMs) between the SDBT and SDCON groups, as well as between the KOBT and SDBT groups. For additional details, refer to Supplementary Table S7.

#### 3.3.3. The Pivotal Role of TRPV4 in Modulating Metabolic Patterns

**Figure 5:**
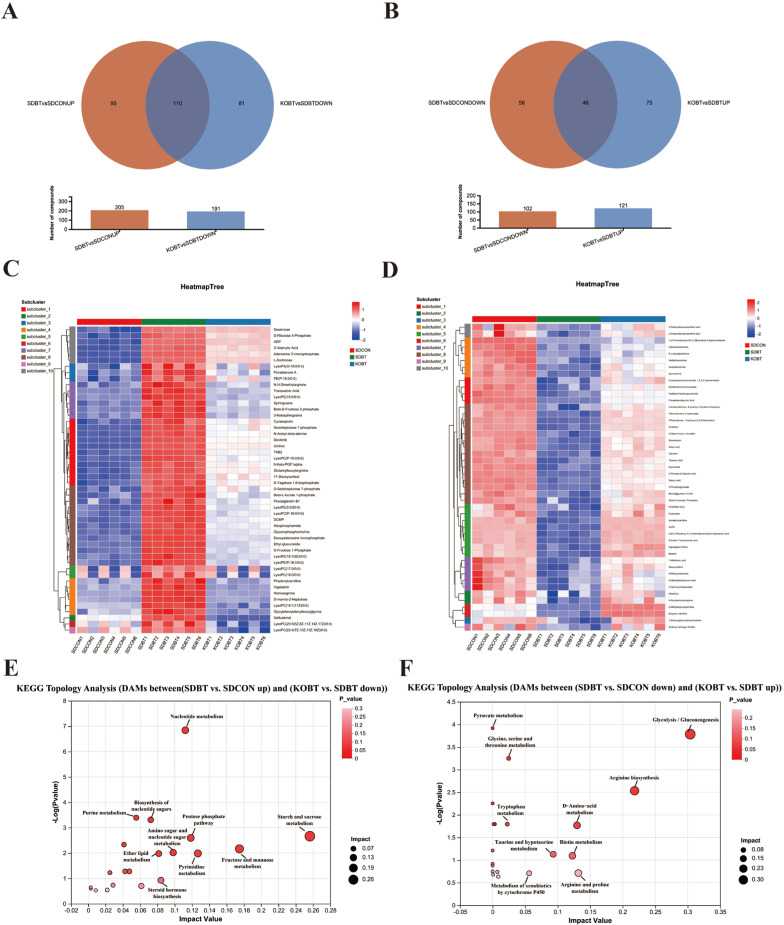
An integrated analysis of DAMs: (**A**) Venn analysis illustrating DAMs suppressed in KOBT versus SDBT alongside those elevated in SDBT compared to SDCON; (**B**) Venn representation displaying DAMs enhanced in KOBT relative to SDBT versus those reduced in SDBT versus SDCON; (**C**) clustered heatmap visualization of DAMs activated through SDBT intervention yet repressed by KOBT treatment; (**D**) heatmap clustering demonstrating DAMs suppressed under SDBT conditions but amplified through KOBT exposure; (**E**) pathway enrichment assessment through KEGG analysis for SDBT-promoted/KOBT-inhibited DAMs; (**F**) KEGG-based metabolic pathway evaluation for SDBT-suppressed/KOBT-enhanced DAMs.

To elucidate the cardioprotective mechanisms of KOBT against SDBT-induced myocardial injury, we systematically analyzed and characterized two distinct metabolite profiles: those exhibiting elevated levels under SDBT exposure but suppressed by KOBT intervention, and conversely, those diminished by SDBT yet enhanced through KOBT administration. Notably, as demonstrated in Figure 5A,B, our analysis revealed 110 metabolites showing SDBT-induced upregulation that were subsequently downregulated by KOBT genetic modification, while 46 metabolites displayed an inverse regulatory pattern. Cluster analysis was subsequently conducted to map the accumulation dynamics of these differentially expressed metabolites across experimental conditions. The resultant clustering patterns corresponding to these two regulatory modes are clearly depicted in Figure 5C,D.

To further investigate the biological functions of these DAMs, we conducted functional enrichment analysis to identify the primary signaling pathways involved. The findings revealed that DAMs characterized by SDBT promotion and KOBT inhibition were predominantly enriched in ether lipid metabolism, fructose and mannose metabolism, the pentose phosphate pathway, nucleotide metabolism, and pyrimidine metabolism (Figure 5E). Additionally, DAMs promoted by SDBT but inhibited by KOBT exhibited significant enrichment in glycolysis, arginine biosynthesis, D-amino-acid metabolism, biotin metabolism, and arginine and proline metabolism pathways (Figure 5F).

### 3.4. Integrated Analysis of the Role of TRPV4 in BCI

**Figure 6:**
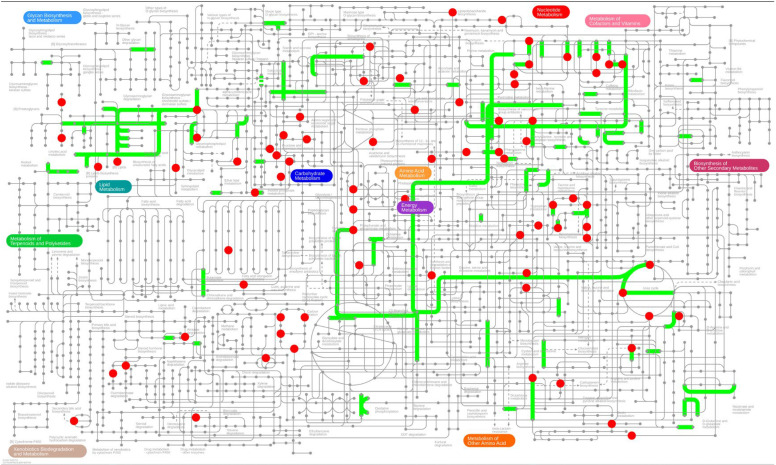
Integrated transcriptome and metabolome analysis in iPath 3.0. The picture represents the pathways for gene set annotation—the green ones represent the pathways for gene annotation in different gene sets, and the red dots represent metabolites, respectively.

To investigate the beneficial effects of the TRPV4 gene on BCI in experimental rats, we conducted a comprehensive integration and detailed investigation of genes showing DEGs and DEMs through the iPath 3.0 analytical platform (http://pathways.embl.de). The results demonstrated that these differential genes and metabolites are significantly enriched in key metabolic pathways such as Glycan Biosynthesis and Metabolism, Lipid Metabolism, Carbohydrate Metabolism, and Amino-Acid Metabolism. Additionally, pathways including “Metabolism,” “Energy Metabolism,” “Nucleotide Metabolism,” and “Metabolism of Cofactors and Vitamins” were identified, as shown in Figure 6. This regulatory influence implies that TRPV4 could mitigate myocardial damage in rodent models by orchestrating multiple biochemical mechanisms, particularly through the modulation of carbohydrate utilization patterns, lipid regulatory networks, energy production cycles, and nucleic acid synthesis pathways.

#### 3.4.1. Effects of TRPV4 on Important Pathways Associated with BCI

**Figure 7:**
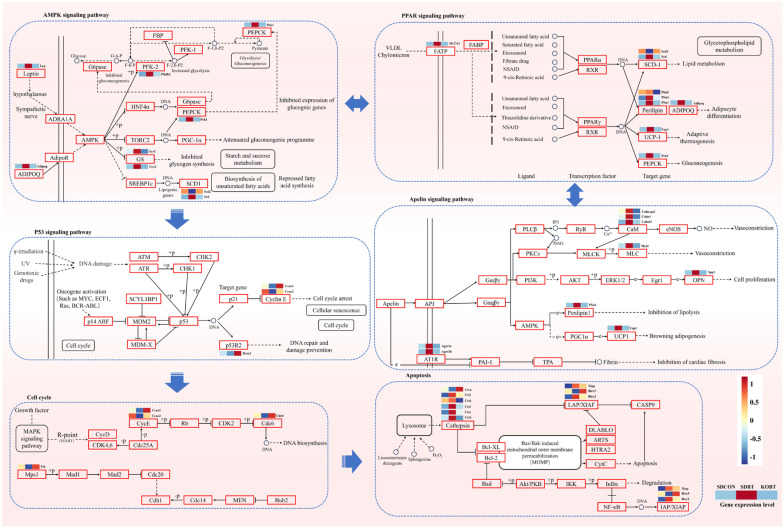
Effect of TRPV4 on BCI response. (The blue dashed boxes represent different pathways, and the genes with colored heatmap bars also represent the key genes regulated by TRPV4.)

As previously discussed, significant enrichment was observed in multiple pathways associated with inflammation and apoptosis. These include the apelin signaling pathway (rno04371), PPAR signaling pathway (rno03320), AMPK signaling pathway (rno04152), p53 signaling pathway (rno04115), cell cycle pathway (rno04110), and apoptosis pathway (rno04210). To further elucidate the mechanism of TRPV4 in BCI, we constructed a pathway enrichment map and heatmap for visual analysis. Compared to the SDBT group, the expression levels of several key genes in these inflammation-related metabolic pathways were significantly upregulated, indicating a pronounced inflammatory response in the heart tissue following BCI. However, the expression of these genes was markedly inhibited in the KOBT group, suggesting that TRPV4 may exert a protective effect against BCI (Figure 7). It is noteworthy that the apelin signaling pathway is closely linked to adipokine signaling and plays a pivotal role in cardiovascular function regulation, energy metabolism, and angiogenesis. In this study, we identified several key regulatory genes, such as Myl4, Ucp1, Calml3, Spp1, Plin1, and Agtr1b, which exhibited significant upregulation or downregulation trends in the SDBT group. Conversely, these trends were reversed in the KOBT group. These findings suggest that these genes may serve as critical regulators in the protective mechanism of TRPV4 against BCI. The complete KEGG pathway annotations are presented in Figure S3.

The PPAR signaling pathway represents a key transcriptional pathway involved in lipid metabolism, inflammatory responses, and cardiovascular disease regulation. Within this pathway, key genes such as Scd, Adipoq, Pck1, Ucp1, Slc27a2, and Plin1 demonstrated significant upregulation or downregulation in the SDBT group, with these changes being reversed in the KOBT group. The full KEGG pathway annotations are illustrated in Figure S4. In the AMPK signaling pathway, important genes including Scd, Adipoq, Gys2, Pck1, Lep, and Pfkfb1 exhibited similar trends (Figure 7 and Figure S5). As downstream targets of the AMPK signaling pathway, the p53 signaling pathway—along with key DEGs in the cell cycle and apoptosis signaling pathways such as Ccne1, Rrm2, Cdc6, Ttk, Ctsw, and Birc5—showed significant upregulation or downregulation in the SDBT group. These changes were notably reversed in the KOBT group (Figure S6–S8). Collectively, these results further substantiate the protective effects of TRPV4 on BCI and its regulatory mechanisms on associated signaling pathways.

#### 3.4.2. Effects of TRPV4 on Pathways Related to BCI Metabolism

**Figure 8:**
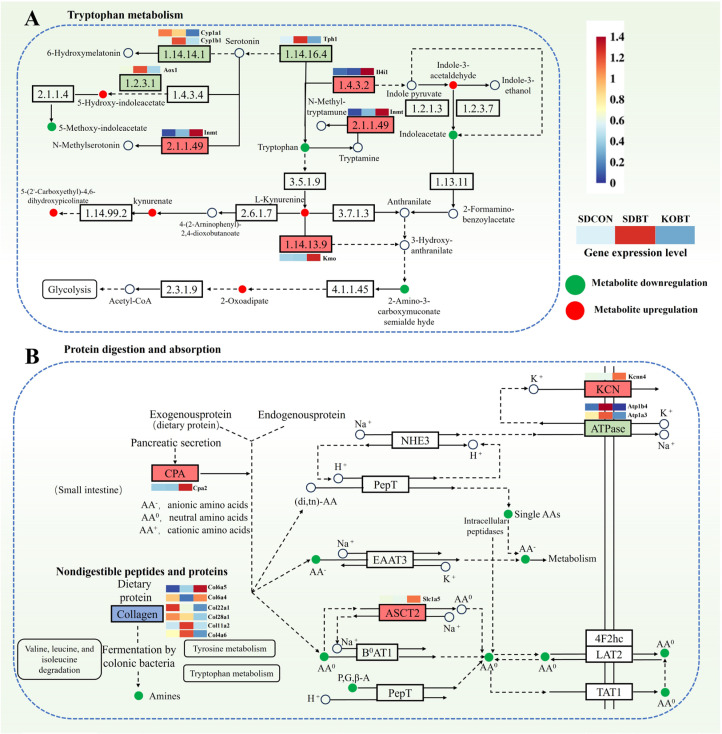
The metabolome transcriptome was combined to analyze the important pathways involved in the effect of TRPV4 on blunt cardiac injury: (**A**) tryptophan metabolism pathway; (**B**) protein digestion and absorption pathway.

In order to further elucidate the mechanism of TRPV4 in BCI, we performed an integrated metabolomic and transcriptomic analysis based on the differential metabolites and genes identified across groups. The results revealed two common pathways shared between the metabolome and transcriptome: tryptophan metabolism (rno00380) and protein digestion and absorption (rno04974) (Figure 8, Figure S9 and S10). The tryptophan metabolism pathway comprises three major branches, namely the kynurenine (Kyn) pathway, the 5-Hydroxytryptamine (5-HT) pathway, and the indole pathway. Studies have demonstrated that dysregulation of tryptophan metabolism plays a critical role in the pathogenesis of various diseases. Through integrative analysis of the metabolome and transcriptome, we identified key metabolites within the tryptophan metabolism pathway, including Lactic Acid, indole-3-carboxaldehyde, l-tryptophan, 2-amino-3-carboxymuconic acid semialdehyde, kynurenine, N-Epsilon-Acetyl-L-Lysine, and 5-hydroxyindoleacetate, as well as their associated genes such as ENSRNOG00000015354 (Aox1), ENSRNOG00000019500 (Cyp1a1), ENSRNOG00000011672 (Tph1), ENSRNOG00000003709 (Kmo), and ENSRNOG00000020148 (Il4i1). These components play pivotal roles in this process. Additionally, the combined analysis indicated that TRPV4 significantly influences the Protein Digestion and Absorption pathway. Specifically, compared to the SDCON group, the SDBT group exhibited significant alterations in the metabolic levels of multiple metabolites involved in protein biosynthesis, such as L-tryptophan, L-tyrosine, L-phenylalanine, L-glutamate, piperidine, and L-proline. Notably, the Protein Digestion and Absorption pathway is a key factor in biosynthesis and includes genes such as ENSRNOG00000024824 (Col22a1), ENSRNOG00000000463 (Col11a2), ENSRNOG00000015948 (Slc1a5), ENSRNOG00000020263 (Atp1a3), ENSRNOG00000024824 (COL22A1), ENSRNOG00000015948 (SLC1A5), and ENSRNOG00000020263 (ATP1A3). These findings further suggest that these key genes and metabolites may serve as important regulators of TRPV4′s protective function in BCI.

### 3.5. qRT-PCR Analysis of Key Genes

**Figure 9:**
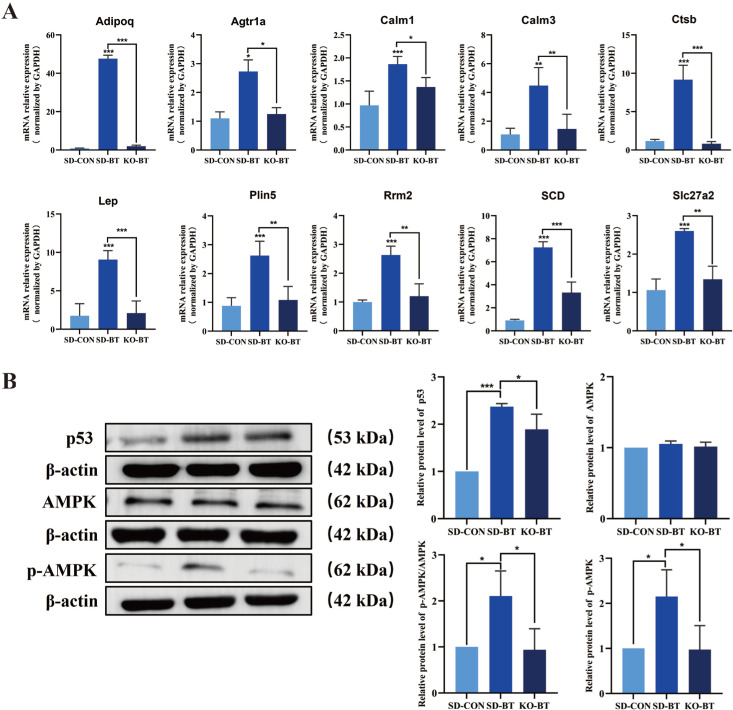
Verification results of relevant genes and proteins: (**A**) A qRT-PCR-based analysis was conducted to assess the expression levels of 10 selected unigenes. (**B**). Western blot analysis results. (Data are expressed as mean ± SD (*n* = 6 for each group). * *p* < 0.05, ** *p* < 0.01, *** *p* < 0.001.)

The qRT-PCR analysis was conducted to confirm the relative expression levels of nine important genes related to PPAR, AMPK, apoptosis, apelin, and the p53 response. The results were consistent with the transcriptome sequencing data, notably, Adipoq, Agtr1a, Calm1, Calm3, Ctsb, Lep, Plin5, Rrm2, SCD, and Slc27a2. (Figure 9A). These findings collectively indicate that the protective role of TRPV4 against BCI is primarily attributed to its modulation of the expression levels of key genes involved in the aforementioned pathways.

### 3.6. Western Blot Analysis

Western blot analysis demonstrated that compared with the SDCON group, the SDBT group exhibited a significant upregulation of p53 expression (*p* < 0.01) and a marked increase in the p-AMPK/AMPK ratio (*p* < 0. 01). In contrast, when compared with the SDBT group, the KOBT group showed a significant decrease in p53 expression (*p* < 0.05) and a notable reduction in the p-AMPK/AMPK ratio (*p* < 0.05) (Figure 9B). These findings suggest that the TRPV4 gene deletion may ameliorate BCI by modulating the p53 and AMPK signaling pathways.

## 4. Discussion

Existing evidence demonstrates that TRPV4 activation exacerbates trauma severity in cardiac injury through distinct mechanisms. Mechanistically, TRPV4-mediated calcium overload has been shown to amplify cardiomyocyte death in response to mechanical stress [13]. In addition, activation of TRPV4 reduces cardiomyocyte tolerance to hypoxia/reoxygenation [14]. Studies demonstrate that TRPV4 channel activation under hypoxia/reoxygenation conditions increases intracellular Ca^2+^ influx, impairs mitochondrial membrane potential, and enhances reactive oxygen species (ROS) production in cardiomyocytes, exacerbating oxidative stress damage. Moreover, TRPV4 loss exhibits a substantial protective effect post-myocardial infarction [7], reducing cardiac fibrosis and preserving cardiomyocyte integrity in the infarct-surrounding area. Thus, an in-depth investigation of TRPV4 gene function and the properties of its encoded protein holds significant implications for elucidating BCI mechanisms and developing therapeutic strategies.

In this study, we compared the inflammatory response and tissue repair between the KOBT and SDBT groups following BCI. Results indicate that KOBT rats exhibited significantly reduced IL-6 and IL-1β expression levels post-injury compared to SDBT rats. Additionally, HE staining revealed markedly diminished cardiac tissue damage and improved myocardial structural integrity in KOBT rats relative to SDBT rats. These findings suggest that the TRPV4 gene is associated with attenuated inflammatory responses and enhanced tissue repair after BCI.

To further explore the potential mechanism underlying TRPV4 gene knockout in BCI, we conducted a cardiac transcriptome analysis. It is worth noting that compared with SDCON, Adipoq, Agtr1a, Calm1, Calm3, Ctsb, Lep, Plin5, Rrm2, SCD, and Slc27a2 were significantly upregulated in the SDBT group. Interestingly, compared with the SDBT group, these nine genes were significantly reversed in the KOBT group. Interestingly, analysis of the STRING database (https://cn.string-db.org/) suggests that there is an interaction network involving Slc27a2, SCD, Lep, and Adipog, as well as between TRPV4 and its potential interacting partners Calm3 and Calm1. Furthermore, examination of the ChIPBase v3.0 database (https://rnasysu.com/chipbase3/index.php, accessed on 4 July 2025) revealed a positive correlation among Slc27a2, Plin5, Lep, Adipog, and the TRPV4 gene in rat heart tissue. These findings are consistent with our experimental results, which indicate that the TRPV4 gene may induce downregulation of the aforementioned genes. These differentially expressed genes may participate in biological processes such as inflammation, apoptosis, and tissue repair post-myocardial injury. Further analysis revealed significant enrichment of these genes in p53, apelin, apoptosis, cell-cycle, AMPK, and PPAR signaling pathways. Consequently, these differentially expressed genes may serve as potential biomarkers for the protective effects of the TRPV4 gene on BCI.

The Slc27a2 gene (solute carrier family 27 member 2) encodes long-chain fatty acid CoA ligase, which plays a role in fatty acid activation and metabolism and is primarily localized in the endoplasmic reticulum and peroxisomes [15]. Studies indicate that azithromycin-induced cardiotoxicity leads to upregulation of Slc27a2 gene expression [16]. This indicates that cardiac dysfunction can lead to high expression of the Slc27a2 gene, and as a special type of cardiac dysfunction, BCI also shows this feature. In this study, we observed significant downregulation of Slc27a2 gene expression following TRPV4 gene knockout, suggesting that TRPV4 loss may influence cardiotoxicity and functional recovery via regulation of Slc27a2 expression.

The SCD gene (stearoyl–CoA desaturase gene) encodes a key enzyme responsible for converting saturated fatty acids into monounsaturated fatty acids, thereby regulating fatty acid composition and metabolism. In the heart, SCD gene expression and activity are closely linked to cardiomyocyte energy metabolism. Research shows that SCD gene upregulation may result in unsaturated and saturated lipid accumulation, exerting detrimental effects on the heart [17]. These metabolic alterations may induce lipid metabolism disorders in cardiomyocytes, potentially affecting cardiac function. In this study, we found that SCD gene expression was significantly downregulated following TRPV4 gene knockout, indicating that TRPV4 loss may protect against cardiac injury by modulating SCD gene expression and improving cardiomyocyte fatty acid metabolism.

Plin5, a member of the Perilipin family proteins, is widely distributed in oxidized tissues such as the heart, liver, skeletal muscle, and brown adipose tissue [18]. Studies demonstrate that Plin5 overexpression promotes cardiac hypertrophy, leading to increased lipid droplet accumulation in cardiomyocytes and impaired mitochondrial function, thereby affecting cardiac systolic function [19]. In this study, we observed significantly downregulated Plin5 gene expression in KOBT rats, suggesting that TRPV4 loss may affect myocardial cell lipid metabolism and oxidative stress response via regulation of Plin5 expression levels, ultimately conferring potential protection to cardiac function.

The Lep gene (leptin gene), secreted by adipocytes, primarily regulates energy balance, appetite, and weight [20]. Research indicates that elevated Lep levels are closely associated with the onset and progression of cardiovascular diseases such as heart failure, hypertension, and coronary heart disease [21]. In this study, we found that KOBT rats exhibited significantly downregulated Lep gene expression, potentially related to reduced cardiac inflammation via inhibition of inflammatory signaling pathways and decreased inflammatory factor release.

The Adipoq gene encodes a hormone secreted by adipocytes with diverse biological functions, including lipid metabolism regulation, insulin resistance improvement, anti-inflammatory properties, and cardiovascular system protection [22]. Studies show that Adipoq levels increase in heart failure (HF) and further elevate as cardiac function deteriorates [23]. In this study, we observed significantly downregulated Adipoq gene expression in KOBT rats, representing an adaptive regulatory mechanism for cardiac injury. This suggests that TRPV4 loss may indirectly affect cardiomyocyte metabolism and the inflammatory response by altering adipose tissue function, thereby playing a potential regulatory role in the pathophysiological process of cardiac injury.

The Ctsb gene encodes a lysosomal cysteine protease that plays a crucial role in intracellular proteolysis and participates in protein turnover, apoptosis, and autophagy [24]. Research demonstrates that high Ctsb expression correlates with severe atherosclerosis, increasing heart failure risk, while Ctsb knockout reduces myocardial cell apoptosis and alleviates stress-overload-induced cardiac remodeling in mice [25,26]. In this study, we found that KOBT rats exhibited significantly downregulated Ctsb gene expression, potentially related to protective mechanisms post-cardiac injury.

The Agtr1a gene plays a vital role in various physiological processes, including blood pressure regulation, cardiovascular system development, cytokine production, and aging in multicellular organisms [27]. Studies show that Agtr1a inhibition not only enhances AML chemotherapy sensitivity but also significantly reduces chemotherapy-induced cardiotoxicity [28]. In this study, we found that KOBT rats exhibited significantly downregulated Agtr1a gene expression, potentially related to reduced cardiomyocyte apoptosis, inflammatory response, and fibrosis, thereby improving cardiac function.

The Calm1 gene, located on chromosome 14q32, is one of three genes encoding calmodulin (CAM) and participates in regulating various cellular processes, including ion channel function, enzyme activity, smooth muscle contraction, the inflammatory response, apoptosis, and the immune response [29]. Research indicates that Calm1 gene knockout may reduce myocardial cell damage via regulation of calcium ion signaling pathways, thus playing a potential protective role in cardiac function [30]. In this study, we found that KOBT rats exhibited significantly downregulated Calm1 gene expression post-BCI, potentially related to calcium homeostasis, apoptosis, gene regulation, the inflammatory response, and metabolic disorders.

The Rrm2 gene plays a critical role in DNA synthesis and cell proliferation, with its abnormal expression closely linked to the occurrence, invasion, and drug resistance of various malignant tumors [31]. Studies show that Rrm2 overexpression can enhance cardiomyocyte contractile function by increasing intracellular dATP levels (approximately tenfold) [32]. In this study, we found that KOBT rats exhibited significantly upregulated Rrm2 gene expression post-BCI, potentially related to improved cardiac function via promotion of DNA synthesis and cell metabolism.

Metabolomics, as a novel omics analysis technology in the post-genomic era, enables efficient and sensitive detection of disease pathological processes or drug metabolism pathways. With advancements in bioinformatics technology, analyzing metabolite changes and their interactions holds great significance for understanding disease pathogenesis [33]. Based on previous findings, this study utilized bioinformatics methods to analyze metabolome changes in rats with BCI and mapped relevant metabolic pathways. A total of 982 metabolites were involved in 156 metabolic pathways. A KEGG topological analysis visually demonstrated the importance of metabolite pathways and their interrelationships with other pathways. Through joint analysis of DEGs and DAMs, two primary metabolic pathways—protein digestion and absorption and tryptophan metabolism—were selected.

Tryptophan, an essential aromatic amino acid, serves not only as a fundamental protein component but also actively participates in various biological metabolic pathways and signal transduction cascades in organisms. Its metabolism primarily occurs through three pathways: the kynurenine pathway, serotonin pathway, and indole pathway [34]. In our study, the deletion of the TRPV4 gene can cause the activation of the tryptophan metabolic pathway. The tryptophan metabolic pathway can be indirectly involved in the proliferation, differentiation, apoptosis, oxidative stress, ubiquitination, and proteasome degradation processes of cardiomyocytes through the Aryl Hydrocarbon Receptor (AHR) (a type of ligand-dependent transcription factor) [35,36]. The activation of AHR during myocardial infarction can regulate immune responses, reduce infarction area, and improve left ventricular ejection fraction [37]. Another study has shown that activating AHR can inhibit myocardial cell apoptosis, thereby alleviating cardiac remodeling and dysfunction in rats with myocardial infarction [38]. In our study, the absence of TRPV4 can lead to a reduction in inflammation, a decrease in the degree of myocardial injury, and activation of the tryptophan metabolic pathway, thereby improving BCI. Interestingly, metabolomic analysis revealed significant changes in multiple metabolites within the tryptophan metabolic pathway in a rat model of BCI. Specifically, metabolites such as 5-hydroxy-indoleacetate, 5-(2’-carboxyethyl)-4,6-dihydroxypicolinate, kynurenate, 2-oxoadipate, indole-3-acetaldehyde, and L-kynurenine exhibited significant upregulation, whereas metabolites such as 5-methoxy-indoleacetate, tryptophan, 2-amino-3-carboxymuconate semialdehyde, and indoleacetate showed significant downregulation. These results suggest that metabolite changes in the tryptophan metabolic pathway play a crucial role in the pathophysiological processes of blunt cardiac injury, with these metabolites serving as potential biomarkers for early diagnosis and providing new targets for targeted therapeutic strategies.

Protein digestion and absorption constitute a key metabolic process for obtaining essential amino acids and maintaining physiological functions. In the digestive tract, proteins are first broken down into peptides by pepsin in the stomach and further decomposed into small peptides and free amino acids by trypsin and chymotrypsin in the small intestine. These small peptides and amino acids enter the bloodstream via intestinal epithelial cell transporters and are transported to tissues and organs throughout the body [39]. Metabolomic studies indicate that the efficiency of protein digestion and absorption pathways not only affects nutrient supply but may also influence systemic metabolic states by regulating metabolite levels, further associating with physiological functions such as cardiovascular health [40]. This study found that in a mouse model of BCI, multiple amino-acid metabolite levels significantly decreased, including anionic amino acids (AA-), neutral amino acids (AA0), and cationic amino acids (AA+). These findings suggest that metabolic changes in protein digestion and absorption pathways may represent an important aspect of systemic metabolic disorders post-cardiac injury, warranting further investigation into their potential effects on cardiovascular health.

In this study, metabonomics and transcriptomics were employed to investigate the critical role of TRPV4 in BCI and its underlying mechanisms, demonstrating that TRPV4 gene deletion significantly reduces BCI severity. Therefore, future investigations into drugs targeting TRPV4 could develop potential therapeutic strategies for BCI and related inflammatory diseases.

While this study elucidates the role of TRPV4 in rat BCI and its underlying mechanisms through integrated transcriptomic and metabolomic analyses, several limitations should be acknowledged. Firstly, rodent models may not fully recapitulate the complex pathophysiology of human BCI, particularly regarding common clinical comorbidities and individual variations. Secondly, the exclusive use of female animals precludes evaluation of potential sex-specific effects in TRPV4-mediated cardioprotection. Additionally, the lack of cellular-level validation using TRPV4 inhibitors represents another constraint. Future investigations should focus on validating TRPV4’s clinical relevance in large animal models; conducting sex-stratified analyses to elucidate potential gender dimorphisms; and employing cardiomyocyte cultures (e.g., H9C2 cells) with pharmacological TRPV4 inhibition to further verify its mechanistic role in BCI. These approaches will facilitate the clinical translation of TRPV4-targeted therapies and provide novel precision medicine strategies for BCI management.

## 5. Conclusions

The serological and histological index results demonstrated that, in comparison to the SDBT group, the extent of myocardial tissue damage was significantly diminished in the KOBT group. Transcriptome analysis indicated that TRPV4 might exert a protective effect on BCI via interactions among the p53, apelin, apoptosis, cell cycle, AMPK, and PPAR signaling pathways. Metabolic investigations further revealed that TRPV4 could modulate multiple metabolic pathways in myocardial tissue, such as nucleotide metabolism, amino-acid metabolism, biotin metabolism, arginine and proline metabolism, the pentose phosphate pathway, and fructose and mannose metabolism. These findings suggest that the TRPV4 gene plays a significant role in BCI. Considering its potential role in cardiac function, TRPV4 could serve as a novel therapeutic target for treating BCI. Nevertheless, despite its promising implications in cardiac research, current studies remain at an early stage. Further fundamental experimental and clinical investigations are required to elucidate its precise mechanism of action and clinical translational potential. Subsequent studies will delve into the exact role of TRPV4 in maintaining heart health and provide a theoretical foundation for the development of innovative TRPV4-based therapies.

## Data Availability

The original contributions presented in this study are included in the article. Further inquiries can be directed to the corresponding author.

## Supplementary Materials

The following supporting information can be downloaded at https://www.mdpi.com/article/10.3390/metabo15080512/s1, Table S1. Primer information of genes used for qPCR validation; Table S2. Sequencing data statistics; Table S3. Results were compared with reference genomes; Table S4. GO results of all the enriched terms of DEGs from the comparison of SDBT versus SDCON; Table S5. GO results of all the enriched terms of DEGs from the comparison of KOBT versus SDBT; Table S6. Total ion count and identification statistics; Table S7. Enrichment of KEGG metabolic pathway of important metabolites; Figure S1. The total ion chromatogram in positive ion mode of cardiac tissues in different groups; Figure S2. The total ion chromatogram in negative ion mode of cardiac tissues in different groups; Figure S3. Enrichment of DEGs in apelin signaling pathway; Figure S4. Enrichment of DEGs in PPAR signaling pathway; Figure S5. Enrichment of DEGs in AMPK signaling pathway; Figure S6. Enrichment of DEGs in P53 signaling pathway; Figure S7. Enrichment of DEGs in cell cycle signaling pathway; Figure S8. Enrichment of DEGs in apoptosis signaling pathway; Figure S9. Enrichment of DEGs and DAMs in tryptophan metabolism pathway; Figure S10. Enrichment of DEGs and DAMs in protein digestion and absorption pathway.
