# Supplementary material for: An Integrated Analysis of Transcriptomics and Metabolomics Elucidates the Role and Mechanism of TRPV4 in Blunt Cardiac Injury
Source: Metabolites. 2025 Jul 31;15(8):512. doi: 10.3390/metabo15080512 (PMC12388018; doi:10.3390/metabo15080512)
Supplement: Supplementary file 1 [file metabolites-15-00512-s001.zip › metabolites-3710499-supplementary.pdf]

# An Integrated Analysis of Transcriptomics and Metabolomics Elucidates the Role and Mechanism of TRPV4 in Blunt Cardiac Injury

**Liancong Gao [1,†], Liu Han [2,†], Xiangyu Ma [2], Huiyan Wang [3], Mutan Li [1] and Jianhui Cai [1,*]**

[1] Clinical Medical College, Jilin Medical University, Jilin132013, China; gaolc@jlmu.edu.cn (L.G.); limutan@jlmu.edu.cn (M.L.)
[2] College of Pharmacy, Jilin Medical University, Jilin132013, China; hanliu@jlmu.edu.cn (L.H.); maxy@jlmu.edu.cn (X.M.)
[3] Jilin Provincial Technology Collaborative Innovation Center of Antibody Engineering, Jilin Medical University, Jilin132013, China; whyan@jlmu.edu.cn
* Correspondence: caijh@jlmu.edu.cn
† Co-first authors: these authors contributed equally to this work.

**Table S1.** Primer information of genes used for qPCR validation.

**Table S2.** Sequencing data statistics.

**Table S3.** Results were compared with reference genomes.

**Table S4.** GO results of all the enriched terms of DEGs from the comparison of SDBT versus SDCON.

**Table S5.** GO results of all the enriched terms of DEGs from the comparison of KOBT versus SDBT.

**Table S6.** Total ion count and identification statistics

**Table S7.** Enrichment of KEGG metabolic pathway of important metabolites.

**Figure S1.** The total ion chromatogram in positive ion mode of cardiac tissues in different groups.

**Figure S2.** The total ion chromatogram in negative ion mode of cardiac tissues in different groups.

**Figure S3.** Enrichment of DEGs in apelin signaling pathway.

**Figure S4.** Enrichment of DEGs in PPAR signaling pathway.

**Figure S5.** Enrichment of DEGs in AMPK signaling pathway.

**Figure S6.** Enrichment of DEGs in P53 signaling pathway.

**Figure S7.** Enrichment of DEGs in cell cycle signaling pathway.

**Figure S8.** Enrichment of DEGs in apoptosis signaling pathway.

**Figure S9.** Enrichment of DEGs and DAMs in tryptophan metabolism pathway.

**Figure S10.** Enrichment of DEGs and DAMs in protein digestion and absorption pathway.

**Table S1.** Primer information of genes used for qPCR validation.

| Primers | Sequence (5' -> 3') | Amplicon Size | Genbank ID |
|---|---|---|---|
| SCD-F | AGTACCGCTGGCACATCAACT | 172 | NM_139192 |
| SCD-R | GGAACAGGAACTCAGAAGCCCA | | |
| PLIN5-F | GGCAAATCAGAGGAGCTGGT | 135 | NM_001134637 |
| PLIN5-R | ACGCACAAAGTAGCCCTGTT | | |

| | | | |
|---|---|---|---|
| Lep-F | GGGTTTCGTGGTGCTGACTA | 156 | NM_013076 |
| Lep-R | TCCTGTTGTTCTGTCCCGTT | | |
| Adipoq-F | TCTGGCTCCAAGTGTATGGG | 161 | NM_144744 |
| Adipoq-R | AGCCTGTCGCCTGTTCTTTG | | |
| Agtr1a-F | TTGAGTCCTGTTCCACCCGA | 187 | NM_030985 |
| Agtr1a-R | CGAAATCCACTTGACCTGGTG | | |
| Ctsb-F | ACACCAATGGCCGAGTCAAT | 90 | NM_022597 |
| Ctsb-R | AGCCACCATTACAGCCATCC | | |
| Calm1-F | GAAATCCGTGAGGCATTCCG | 180 | NM_031969 |
| Calm1-R | CTGTACGAATTCTTCATAGTTGACC | | |
| Rrm2-F | GCCGAGCTGGAAAGTAAAGC | 90 | NM_001025740 |
| Rrm2-R | GGGAAAGACAACGAAACGGC | | |
| Calm3-F | AATGAGGTGGATGCTGATGGCAA | 100 | NM_012518 |
| Calm3-R | GTATCTCCTCCTCGCTGTCTGT | | |
| GAPDH-F | GGAGTAAGAAACCCTGGACCAC | 185 | NM_017008 |
| GAPDH-R | GATGGTATTCGAGAGAAGGGAGGG | | |
| Slc27a2-F | CATGATTGGCCTCCACGGAT | 139 | NM_031736 |
| Slc27a2-R | GTACCGAAGCAGTTCACCGA | | |

**Table S2.** Sequencing data statistics.

| Sample | Raw reads | Raw bases | Clean reads | Clean bases | Error rate (%) | Q20(%) | Q30(%) | GC content (%) |
|---|---|---|---|---|---|---|---|---|

| | | | | | | | | |
|---|---|---|---|---|---|---|---|---|
| SDCON1 | 41364694 | 6246068794 | 41009384 | 6140708014 | 0.0125 | 98.38 | 95.11 | 47.24 |
| SDCON2 | 51659496 | 7800583896 | 51082716 | 7657887100 | 0.0121 | 98.67 | 95.95 | 47.3 |
| SDCON3 | 54013152 | 8155985952 | 53418230 | 8005071355 | 0.0121 | 98.65 | 95.84 | 47.18 |
| SDCON4 | 54821964 | 8278116564 | 54198866 | 8118559485 | 0.0121 | 98.68 | 95.96 | 47.27 |
| SDCON5 | 55309804 | 8351780404 | 54687392 | 8178889276 | 0.012 | 98.7 | 96.04 | 47.09 |
| SDCON6 | 51804274 | 7822445374 | 51214960 | 7665644103 | 0.0121 | 98.67 | 95.95 | 47.22 |
| SDBT1 | 49310360 | 7445864360 | 48763074 | 7301938910 | 0.012 | 98.69 | 95.99 | 47.54 |
| SDBT2 | 49040718 | 7405148418 | 48502572 | 7265134747 | 0.0121 | 98.67 | 95.94 | 47.45 |
| SDBT3 | 49059148 | 7407931348 | 48500942 | 7256422607 | 0.0121 | 98.65 | 95.86 | 47.45 |
| SDBT4 | 50174860 | 7576403860 | 49610444 | 7431072948 | 0.0121 | 98.68 | 95.97 | 47.44 |
| SDBT5 | 47059388 | 7105967588 | 46311342 | 6940923279 | 0.0132 | 98.04 | 93.9 | 47.63 |
| SDBT6 | 46227992 | 6980426792 | 45711880 | 6856748963 | 0.0121 | 98.68 | 95.95 | 47.6 |
| KOBT1 | 48224846 | 7281951746 | 47705694 | 7154829888 | 0.0121 | 98.68 | 95.95 | 47.1 |
| KOBT2 | 45584784 | 6883302384 | 45108006 | 6766902533 | 0.012 | 98.69 | 95.99 | 47.11 |
| KOBT3 | 46643716 | 7043201116 | 46130182 | 6907898779 | 0.0121 | 98.67 | 95.92 | 47.05 |
| KOBT4 | 45602178 | 6885928878 | 45101868 | 6774512733 | 0.0121 | 98.68 | 95.96 | 46.76 |
| KOBT5 | 49296978 | 7443843678 | 48752768 | 7315640321 | 0.0121 | 98.66 | 95.9 | 47.24 |
| KOBT6 | 53011552 | 8004744352 | 52423276 | 7858896102 | 0.0121 | 98.67 | 95.92 | 47.32 |

**Table S3.** Results were compared with reference genomes.

| Sample | Total reads | Total mapped | Multiple mapped | Unique mapped |
|---|---|---|---|---|
| SDCON1 | 41009384 | 39920183(97.34%) | 1296863(3.16%) | 38623320(94.18%) |
| SDCON2 | 51082716 | 49787620(97.46%) | 2057232(4.03%) | 47730388(93.44%) |
| SDCON3 | 53418230 | 52071514(97.48%) | 2062364(3.86%) | 50009150(93.62%) |
| SDCON4 | 54198866 | 52827575(97.47%) | 2119270(3.91%) | 50708305(93.56%) |
| SDCON5 | 54687392 | 53322148(97.5%) | 2162725(3.95%) | 51159423(93.55%) |
| SDCON6 | 51214960 | 49913080(97.46%) | 2007885(3.92%) | 47905195(93.54%) |
| SDBT1 | 48763074 | 47592261(97.6%) | 1895123(3.89%) | 45697138(93.71%) |
| SDBT2 | 48502572 | 47341650(97.61%) | 1864247(3.84%) | 45477403(93.76%) |
| SDBT3 | 48500942 | 47278903(97.48%) | 1864785(3.84%) | 45414118(93.64%) |
| SDBT4 | 49610444 | 48461855(97.68%) | 1942632(3.92%) | 46519223(93.77%) |
| SDBT5 | 46311342 | 44973970(97.11%) | 1806184(3.9%) | 43167786(93.21%) |
| SDBT6 | 45711880 | 44590421(97.55%) | 1782480(3.9%) | 42807941(93.65%) |
| KOCON1 | 46635930 | 45487637(97.54%) | 1811329(3.88%) | 43676308(93.65%) |
| KOCON2 | 53253218 | 51955229(97.56%) | 1986458(3.73%) | 49968771(93.83%) |
| KOCON3 | 50010690 | 48776598(97.53%) | 2027368(4.05%) | 46749230(93.48%) |
| KOCON4 | 57956940 | 56468555(97.43%) | 2303487(3.97%) | 54165068(93.46%) |
| KOCON5 | 49568076 | 48370264(97.58%) | 1934840(3.9%) | 46435424(93.68%) |
| KOCON6 | 47860390 | 46628959(97.43%) | 1870381(3.91%) | 44758578(93.52%) |

**Table S4.** GO results of all the enriched terms of DEGs from the comparison of SDBT versus SDCON.

| GO id | Term description | Term type | SDBT_vs_SDCON number | SDBT_vs_SDCON percent |
|---|---|---|---|---|
| GO:0001906 | cell killing | biological_process | 1 | 1/ 314 |
| GO:0002376 | immune system process | biological_process | 16 | 16/ 314 |
| GO:0065007 | biological regulation | biological_process | 140 | 140/ 314 |
| GO:0008152 | metabolic process | biological_process | 73 | 73/ 314 |
| GO:0098743 | cell aggregation | biological_process | 2 | 2/ 314 |
| GO:0051704 | multi-organism process | biological_process | 10 | 10/ 314 |
| GO:0040011 | locomotion | biological_process | 17 | 17/ 314 |
| GO:0022414 | reproductive process | biological_process | 26 | 26/ 314 |
| GO:0008283 | cell population proliferation | biological_process | 11 | 11/ 314 |
| GO:0071840 | cellular component organization or biogenesis | biological_process | 55 | 55/ 314 |
| GO:0009987 | cellular process | biological_process | 154 | 154/ 314 |
| GO:0032502 | developmental process | biological_process | 59 | 59/ 314 |
| GO:0032501 | multicellular organismal process | biological_process | 47 | 47/ 314 |
| GO:0040007 | growth | biological_process | 4 | 4/ 314 |
| GO:0048511 | rhythmic process | biological_process | 5 | 5/ 314 |
| GO:0051179 | localization | biological_process | 30 | 30/ 314 |
| GO:0022610 | biological adhesion | biological_process | 8 | 8/ 314 |

| | | | | |
|---|---|---|---|---|
| GO:0007610 | behavior | biological_process | 10 | 10/ 314 |
| GO:0023052 | signaling | biological_process | 7 | 7/ 314 |
| GO:0050896 | response to stimulus | biological_process | 67 | 67/ 314 |
| GO:0031974 | membrane-enclosed lumen | cellular_component | 2 | 2/ 314 |
| GO:0032991 | protein-containing complex | cellular_component | 54 | 54/ 314 |
| GO:0044456 | synapse part | cellular_component | 15 | 15/ 314 |
| GO:0005623 | cell | cellular_component | 2 | 2/ 314 |
| GO:0044425 | membrane part | cellular_component | 87 | 87/ 314 |
| GO:0044421 | extracellular region part | cellular_component | 35 | 35/ 314 |
| GO:0044422 | organelle part | cellular_component | 73 | 73/ 314 |
| GO:0043226 | organelle | cellular_component | 94 | 94/ 314 |
| GO:0045202 | synapse | cellular_component | 12 | 12/ 314 |
| GO:0016020 | membrane | cellular_component | 62 | 62/ 314 |
| GO:0030054 | cell junction | cellular_component | 12 | 12/ 314 |
| GO:0005576 | extracellular region | cellular_component | 9 | 9/ 314 |
| GO:0044217 | other organism part | cellular_component | 1 | 1/ 314 |
| GO:0044464 | cell part | cellular_component | 172 | 172/ 314 |
| GO:0099080 | supramolecular complex | cellular_component | 7 | 7/ 314 |
| GO:0045182 | translation regulator activity | molecular_function | 1 | 1/ 314 |
| GO:0140110 | transcription regulator activity | molecular_function | 13 | 13/ 314 |
| GO:0005198 | structural molecule activity | molecular_function | 17 | 17/ 314 |
| GO:0038024 | cargo receptor activity | molecular_function | 2 | 2/ 314 |

| | | | | |
|---|---|---|---|---|
| GO:0016209 | antioxidant activity | molecular_function | 1 | 1/ 314 |
| GO:0005215 | transporter activity | molecular_function | 16 | 16/ 314 |
| GO:0098772 | molecular function regulator | molecular_function | 22 | 22/ 314 |
| GO:0005488 | binding | molecular_function | 143 | 143/ 314 |
| GO:0060089 | molecular transducer activity | molecular_function | 20 | 20/ 314 |
| GO:0003824 | catalytic activity | molecular_function | 62 | 62/ 314 |

**Table S5.** GO results of all the enriched terms of DEGs from the comparison of KOBT versus SDBT.

| GO id | Term description | Term type | KOBT_vs_SDBT number | KOBT_vs_SDBT percent |
|---|---|---|---|---|
| GO:0001906 | cell killing | biological_process | 1 | 1/ 643 |
| GO:0002376 | immune system process | biological_process | 35 | 35/ 643 |
| GO:0065007 | biological regulation | biological_process | 308 | 308/ 643 |
| GO:0008152 | metabolic process | biological_process | 141 | 141/ 643 |
| GO:0043473 | pigmentation | biological_process | 1 | 1/ 643 |
| GO:0051704 | multi-organism process | biological_process | 40 | 40/ 643 |
| GO:0040011 | locomotion | biological_process | 26 | 26/ 643 |
| GO:0022414 | reproductive process | biological_process | 42 | 42/ 643 |
| GO:0008283 | cell population proliferation | biological_process | 12 | 12/ 643 |
| GO:0071840 | cellular component organization or biogenesis | biological_process | 117 | 117/ 643 |
| GO:0009987 | cellular process | biological_process | 344 | 344/ 643 |
| GO:0032502 | developmental process | biological_process | 182 | 182/ 643 |

| | | | | |
|---|---|---|---|---|
| GO:0032501 | multicellular organismal process | biological_process | 122 | 122/ 643 |
| GO:0040007 | growth | biological_process | 11 | 11/ 643 |
| GO:0048511 | rhythmic process | biological_process | 10 | 10/ 643 |
| GO:0051179 | localization | biological_process | 100 | 100/ 643 |
| GO:0022610 | biological adhesion | biological_process | 42 | 42/ 643 |
| GO:0007610 | behavior | biological_process | 37 | 37/ 643 |
| GO:0098754 | detoxification | biological_process | 1 | 1/ 643 |
| GO:0023052 | signaling | biological_process | 17 | 17/ 643 |
| GO:0019740 | nitrogen utilization | biological_process | 0 | 0 |
| GO:0050896 | response to stimulus | biological_process | 148 | 148/ 643 |
| GO:0031974 | membrane-enclosed lumen | cellular_component | 4 | 4/ 643 |
| GO:0032991 | protein-containing complex | cellular_component | 98 | 98/ 643 |
| GO:0044456 | synapse part | cellular_component | 51 | 51/ 643 |
| GO:0005623 | cell | cellular_component | 3 | 3/ 643 |
| GO:0044425 | membrane part | cellular_component | 245 | 245/ 643 |
| GO:0044421 | extracellular region part | cellular_component | 94 | 94/ 643 |
| GO:0044422 | organelle part | cellular_component | 145 | 145/ 643 |
| GO:0043226 | organelle | cellular_component | 185 | 185/ 643 |
| GO:0045202 | synapse | cellular_component | 39 | 39/ 643 |
| GO:0016020 | membrane | cellular_component | 172 | 172/ 643 |
| GO:0030054 | cell junction | cellular_component | 34 | 34/ 643 |
| GO:0005576 | extracellular region | cellular_component | 23 | 23/ 643 |

| | | | | |
|---|---|---|---|---|
| GO:0044217 | other organism part | cellular_component | 0 | 0 |
| GO:0044464 | cell part | cellular_component | 382 | 382/ 643 |
| GO:0099080 | supramolecular complex | cellular_component | 17 | 17/ 643 |
| GO:0045182 | translation regulator activity | molecular_function | 3 | 3/ 643 |
| GO:0140110 | transcription regulator activity | molecular_function | 27 | 27/ 643 |
| GO:0005198 | structural molecule activity | molecular_function | 28 | 28/ 643 |
| GO:0044183 | protein folding chaperone | molecular_function | 2 | 2/ 643 |
| GO:0038024 | cargo receptor activity | molecular_function | 5 | 5/ 643 |
| GO:0016209 | antioxidant activity | molecular_function | 2 | 2/ 643 |
| GO:0140313 | molecular sequestering activity | molecular_function | 0 | 0 |
| GO:0005215 | transporter activity | molecular_function | 53 | 53/ 643 |
| GO:0098772 | molecular function regulator | molecular_function | 45 | 45/ 643 |
| GO:0140299 | small molecule sensor activity | molecular_function | 1 | 1/ 643 |
| GO:0005488 | binding | molecular_function | 357 | 357/ 643 |
| GO:0060089 | molecular transducer activity | molecular_function | 48 | 48/ 643 |
| GO:0003824 | catalytic activity | molecular_function | 162 | 162/ 643 |

**Table S6.** Total ion count and identification statistics

| Ion mode | All peaks | Identified metabolites | Metabolites in Library | Metabolites in KEGG |
|---|---|---|---|---|
| pos | 2743 | 531 | 495 | 300 |
| neg | 4448 | 507 | 487 | 293 |

Note: Identified metabolites: the number of metabolites that are ultimately identified through primary and secondary mass spectrometry data and search databases (self-built databases, Metlin, HMDB, etc.); Metabolites in library: Annotate the number of metabolites into public databases such as HMDB and Lipidmaps; Metabolites in KEGG: Annotate the number of metabolites in KEGG database.

**Table S7.** Enrichment of KEGG metabolic pathway of important metabolites.

| Number | First Category | Second Category | Pathway ID | Pathway Description | Enrich Factor | P value |
|---|---|---|---|---|---|---|
| 1 | Human Diseases | Cancer: specific types | map05224 | Breast cancer | 0.333333 | 0.02829 |
| 1 | Environmental Information Processing | Signal transduction | map04068 | FoxO signaling pathway | 0.2 | 0.04671 |
| 1 | Cellular Processes | Cell growth and death | map04114 | Oocyte meiosis | 0.25 | 0.03754 |
| 1 | Cellular Processes | Cell growth and death | map04210 | Apoptosis | 0.25 | 0.03754 |
| 1 | Cellular Processes | Transport and catabolism | map04142 | Lysosome | 0.25 | 0.03754 |
| 1 | Organismal Systems | Endocrine system | map04914 | Progesterone-mediated oocyte maturation | 0.25 | 0.03754 |
| 1 | Human Diseases | Cancer: specific types | map05215 | Prostate cancer | 0.090909 | 0.09996 |
| 1 | Organismal Systems | Endocrine system | map04917 | Prolactin signaling pathway | 0.090909 | 0.09996 |
| 1 | Organismal Systems | Endocrine system | map04614 | Renin-angiotensin system | 0.090909 | 0.09996 |
| 1 | Organismal Systems | Immune system | map04613 | Neutrophil extracellular trap formation | 0.076923 | 0.117 |
| 1 | Cellular Processes | Cell growth and death | map04217 | Necroptosis | 0.1 | 0.09129 |
| 1 | Human Diseases | Neurodegenerative disease | map05014 | Amyotrophic lateral sclerosis | 0.071429 | 0.1255 |

| | | | | | | |
|---|---|---|---|---|---|---|
| 1 | Organismal Systems | Immune system | map04611 | Platelet activation | 0.071429 | 0.1255 |
| 1 | Environmental Information Processing | Signal transduction | map04071 | Sphingolipid signaling pathway | 0.066667 | 0.1338 |
| 1 | Metabolism | Energy metabolism | map00190 | Oxidative phosphorylation | 0.0625 | 0.1421 |
| 1 | Organismal Systems | Endocrine system | map04924 | Renin secretion | 0.058824 | 0.1503 |
| 1 | Human Diseases | Drug resistance: antineoplastic | map01523 | Antifolate resistance | 0.058824 | 0.1503 |
| 1 | Human Diseases | Endocrine and metabolic disease | map04931 | Insulin resistance | 0.052632 | 0.1665 |
| 1 | Organismal Systems | Endocrine system | map04925 | Aldosterone synthesis and secretion | 0.045455 | 0.1901 |
| 1 | Organismal Systems | Environmental adaptation | map04714 | Thermogenesis | 0.043478 | 0.1979 |
| 1 | Metabolism | Metabolism of cofactors and vitamins | map00740 | Riboflavin metabolism | 0.041667 | 0.2055 |
| 1 | Organismal Systems | Endocrine system | map04913 | Ovarian steroidogenesis | 0.041667 | 0.2055 |
| 1 | Organismal Systems | Digestive system | map04973 | Carbohydrate digestion and absorption | 0.037037 | 0.2281 |
| 1 | Human Diseases | Neurodegenerative disease | map05012 | Parkinson disease | 0.038462 | 0.2207 |
| 1 | Organismal Systems | Endocrine system | map04922 | Glucagon signaling pathway | 0.038462 | 0.2207 |
| 1 | Human Diseases | Cancer: overview | map05207 | Chemical carcinogenesis - receptor activation | 0.034483 | 0.2429 |
| 1 | Metabolism | Energy metabolism | map00920 | Sulfur metabolism | 0.030303 | 0.2715 |
| 1 | Human Diseases | Cancer: overview | map05200 | Pathways in cancer | 0.032258 | 0.2573 |
| 1 | Metabolism | Metabolism of other amino acids | map00410 | beta-Alanine metabolism | 0.03125 | 0.2644 |
| 1 | Metabolism | Carbohydrate metabolism | map00620 | Pyruvate metabolism | 0.03125 | 0.2644 |
| 1 | Human Diseases | Neurodegenerative disease | map05022 | Pathways of neurodegeneration - multiple diseases | 0.03125 | 0.2644 |
| 1 | Metabolism | Amino acid metabolism | map00400 | Phenylalanine, tyrosine and tryptophan biosynthesis | 0.028571 | 0.2854 |
| 1 | Metabolism | Lipid metabolism | map00600 | Sphingolipid metabolism | 0.028571 | 0.2854 |

| | | | | | | |
|---|---|---|---|---|---|---|
| 1 | Human Diseases | Cancer: overview | map05230 | Central carbon metabolism in cancer | 0.027027 | 0.299 |
| 1 | Human Diseases | Cardiovascular disease | map05415 | Diabetic cardiomyopathy | 0.025641 | 0.3124 |
| 1 | Metabolism | Lipid metabolism | map00062 | Fatty acid elongation | 0.025 | 0.319 |
| 1 | Metabolism | Lipid metabolism | map00071 | Fatty acid degradation | 0.02 | 0.3817 |
| 1 | Metabolism | Carbohydrate metabolism | map00040 | Pentose and glucuronate interconversions | 0.017241 | 0.4278 |
| 1 | Metabolism | Amino acid metabolism | map00270 | Cysteine and methionine metabolism | 0.014925 | 0.4756 |
| 1 | Metabolism | Xenobiotics biodegradation and metabolism | map00982 | Drug metabolism - cytochrome P450 | 0.011494 | 0.5683 |
| 1 | Organismal Systems | Digestive system | map04976 | Bile secretion | 0.010309 | 0.6085 |
| 2 | Human Diseases | Endocrine and metabolic disease | map04934 | Cushing syndrome | 0.153846 | 0.006458 |
| 2 | Organismal Systems | Endocrine system | map04927 | Cortisol synthesis and secretion | 0.166667 | 0.005498 |
| 2 | Environmental Information Processing | Signal transduction | map04152 | AMPK signaling pathway | 0.090909 | 0.01812 |
| 2 | Metabolism | Lipid metabolism | map00565 | Ether lipid metabolism | 0.08 | 0.02311 |
| 2 | Organismal Systems | Sensory system | map04742 | Taste transduction | 0.0625 | 0.03664 |
| 2 | Organismal Systems | Nervous system | map04726 | Serotonergic synapse | 0.047619 | 0.05994 |
| 2 | Environmental Information Processing | Signaling molecules and interaction | map04080 | Neuroactive ligand-receptor interaction | 0.037736 | 0.08991 |
| 2 | Metabolism | Amino acid metabolism | map00310 | Lysine degradation | 0.035714 | 0.09872 |
| 2 | Metabolism | Lipid metabolism | map00140 | Steroid hormone biosynthesis | 0.020202 | 0.2426 |
| 2 | Metabolism | Global and overview maps | map01240 | Biosynthesis of cofactors | 0.006079 | 0.8318 |
| 3 | Metabolism | Carbohydrate metabolism | map00500 | Starch and sucrose metabolism | 0.081081 | 0.004982 |
| 3 | Metabolism | Carbohydrate metabolism | map00052 | Galactose metabolism | 0.065217 | 0.009172 |

| | | | | | | |
|---|---|---|---|---|---|---|
| 3 | Metabolism | Nucleotide metabolism | map00240 | Pyrimidine metabolism | 0.046875 | 0.02237 |
| 3 | Metabolism | Carbohydrate metabolism | map00030 | Pentose phosphate pathway | 0.054054 | 0.04776 |
| 3 | Metabolism | Carbohydrate metabolism | map00520 | Amino sugar and nucleotide sugar metabolism | 0.02521 | 0.1029 |
| 3 | Environmental Information Processing | Membrane transport | map02010 | ABC transporters | 0.021739 | 0.1427 |
| 3 | Metabolism | Amino acid metabolism | map00380 | Tryptophan metabolism | 0.024096 | 0.1865 |
| 4 | Metabolism | Carbohydrate metabolism | map00051 | Fructose and mannose metabolism | 0.071429 | 0.001825 |
| 4 | Metabolism | Lipid metabolism | map00590 | Arachidonic acid metabolism | 0.050633 | 0.006372 |
| 7 | Metabolism | Global and overview maps | map01250 | Biosynthesis of nucleotide sugars | 0.029851 | 0.01108 |
| 8 | Metabolism | Global and overview maps | map01232 | Nucleotide metabolism | 0.12069 | 8.92E-07 |
| 8 | Human Diseases | Cancer: overview | map05231 | Choline metabolism in cancer | 0.181818 | 0.004609 |
| 8 | Metabolism | Lipid metabolism | map00564 | Glycerophospholipid metabolism | 0.035714 | 0.09872 |
| 9 | Metabolism | Nucleotide metabolism | map00230 | Purine metabolism | 0.069307 | 3.77E-05 |
| 1 | Human Diseases | Cancer: specific types | map05224 | Breast cancer | 0.333333 | 0.02829 |
| 1 | Organismal Systems | Nervous system | map04726 | Serotonergic synapse | 0.02381 | 0.155 |
| 1 | Metabolism | Xenobiotics biodegradation and metabolism | map00980 | Metabolism of xenobiotics by cytochrome P450 | 0.008264 | 0.3871 |
| 1 | Metabolism | Metabolism of other amino acids | map00470 | D-Amino acid metabolism | 0.028986 | 0.03004 |
| 1 | Metabolism | Metabolism of other amino acids | map00480 | Glutathione metabolism | 0.026316 | 0.1413 |
| 1 | Metabolism | Metabolism of other amino acids | map00440 | Phosphonate and phosphinate metabolism | 0.017857 | 0.2014 |
| 1 | Metabolism | Biosynthesis of other secondary metabolites | map00232 | Caffeine metabolism | 0.045455 | 0.08427 |
| 1 | Human Diseases | Cancer: overview | map05208 | Chemical carcinogenesis - reactive oxygen species | 0.017544 | 0.2046 |

| | | | | | | |
|---|---|---|---|---|---|---|
| 1 | Human Diseases | Cancer: overview | map05204 | Chemical carcinogenesis - DNA adducts | 0.012821 | 0.2695 |
| 1 | Metabolism | Amino acid metabolism | map00400 | Phenylalanine, tyrosine and tryptophan biosynthesis | 0.028571 | 0.1309 |
| 1 | Metabolism | Amino acid metabolism | map00330 | Arginine and proline metabolism | 0.014493 | 0.2424 |
| 1 | Metabolism | Carbohydrate metabolism | map00020 | Citrate cycle (TCA cycle) | 0.05 | 0.07689 |
| 1 | Metabolism | Metabolism of other amino acids | map00430 | Taurine and hypotaurine metabolism | 0.041667 | 0.09158 |
| 1 | Organismal Systems | Excretory system | map04964 | Proximal tubule bicarbonate reclamation | 0.058824 | 0.06573 |
| 1 | Metabolism | Carbohydrate metabolism | map00053 | Ascorbate and aldarate metabolism | 0.017544 | 0.2046 |
| 1 | Metabolism | Nucleotide metabolism | map00230 | Purine metabolism | 0.009901 | 0.3348 |
| 1 | Metabolism | Nucleotide metabolism | map00240 | Pyrimidine metabolism | 0.015625 | 0.2269 |
| 1 | Metabolism | Amino acid metabolism | map00270 | Cysteine and methionine metabolism | 0.014925 | 0.2362 |
| 2 | Metabolism | Lipid metabolism | map00561 | Glycerolipid metabolism | 0.052632 | 0.009683 |
| 2 | Metabolism | Carbohydrate metabolism | map00630 | Glyoxylate and dicarboxylate metabolism | 0.03125 | 0.02612 |
| 2 | Metabolism | Metabolism of cofactors and vitamins | map00780 | Biotin metabolism | 0.034483 | 0.1096 |
| 2 | Metabolism | Carbohydrate metabolism | map00030 | Pentose phosphate pathway | 0.054054 | 0.009195 |
| 2 | Metabolism | Global and overview maps | map01232 | Nucleotide metabolism | 0.034483 | 0.02172 |
| 2 | Metabolism | Amino acid metabolism | map00380 | Tryptophan metabolism | 0.024096 | 0.04218 |
| 2 | Metabolism | Amino acid metabolism | map00220 | Arginine biosynthesis | 0.086957 | 0.00361 |
| 3 | Organismal Systems | Endocrine system | map04922 | Glucagon signaling pathway | 0.115385 | 0.00013 |
| 3 | Human Diseases | Cancer: overview | map05230 | Central carbon metabolism in cancer | 0.081081 | 0.000379 |

| | | | | | | |
|---|---|---|---|---|---|---|
| 3 | Metabolism | Amino acid metabolism | map00260 | Glycine, serine and threonine metabolism | 0.0625 | 0.000821 |
| 3 | Metabolism | Carbohydrate metabolism | map00010 | Glycolysis / Gluconeogenesis | 0.09375 | 0.000245 |
| 3 | Metabolism | Carbohydrate metabolism | map00620 | Pyruvate metabolism | 0.09375 | 0.000245 |
| 4 | Environmental Information Processing | Membrane transport | map02010 | ABC transporters | 0.028986 | 0.001827 |
| 4 | Metabolism | Global and overview maps | map01240 | Biosynthesis of cofactors | 0.009119 | 0.1387 |

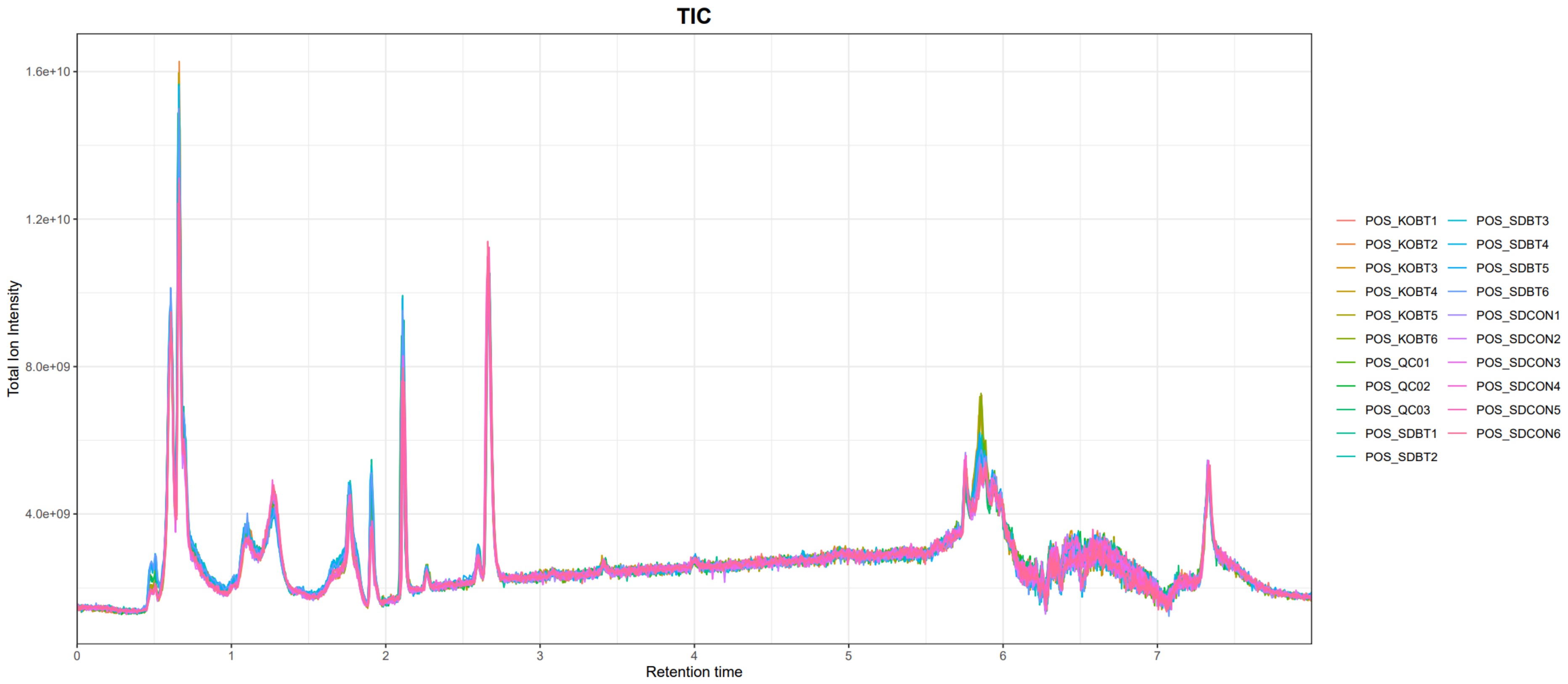


**Figure S1.** The total ion chromatogram in positive ion mode of cardiac tissues in different groups.

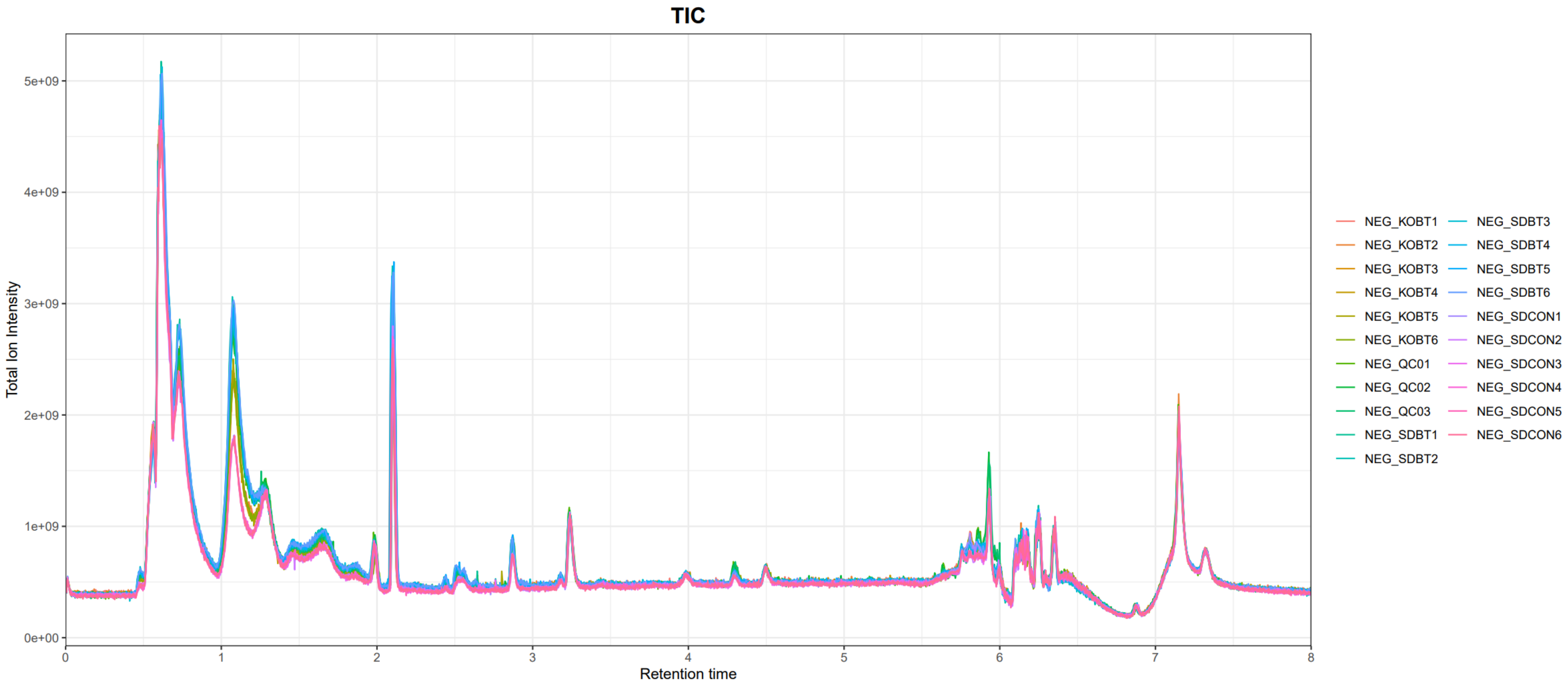


**Figure S2.** The total ion chromatogram in negative ion mode of cardiac tissues in different groups.

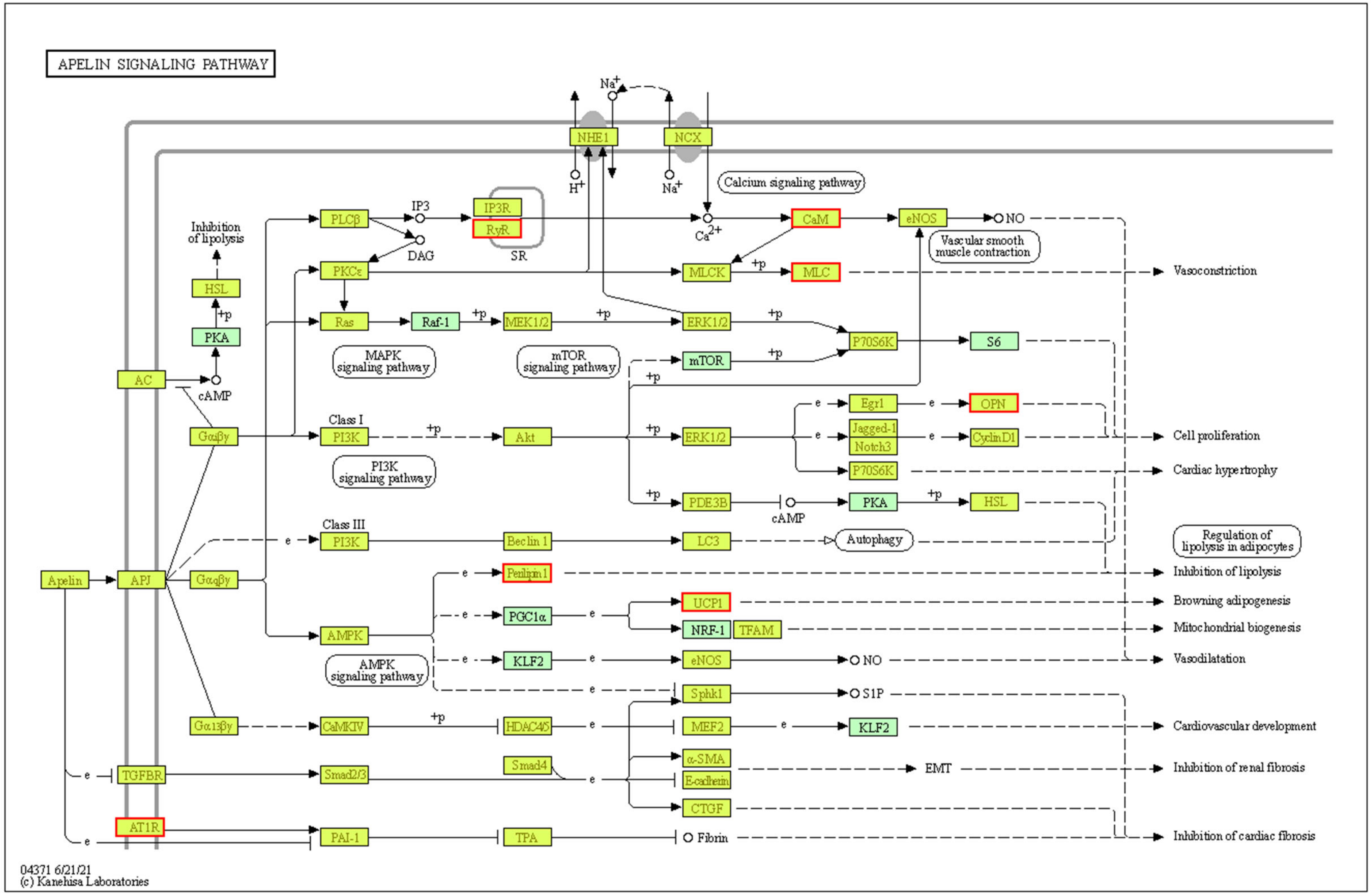


**Figure S3.** Enrichment of DEGs in apelin signaling pathway.

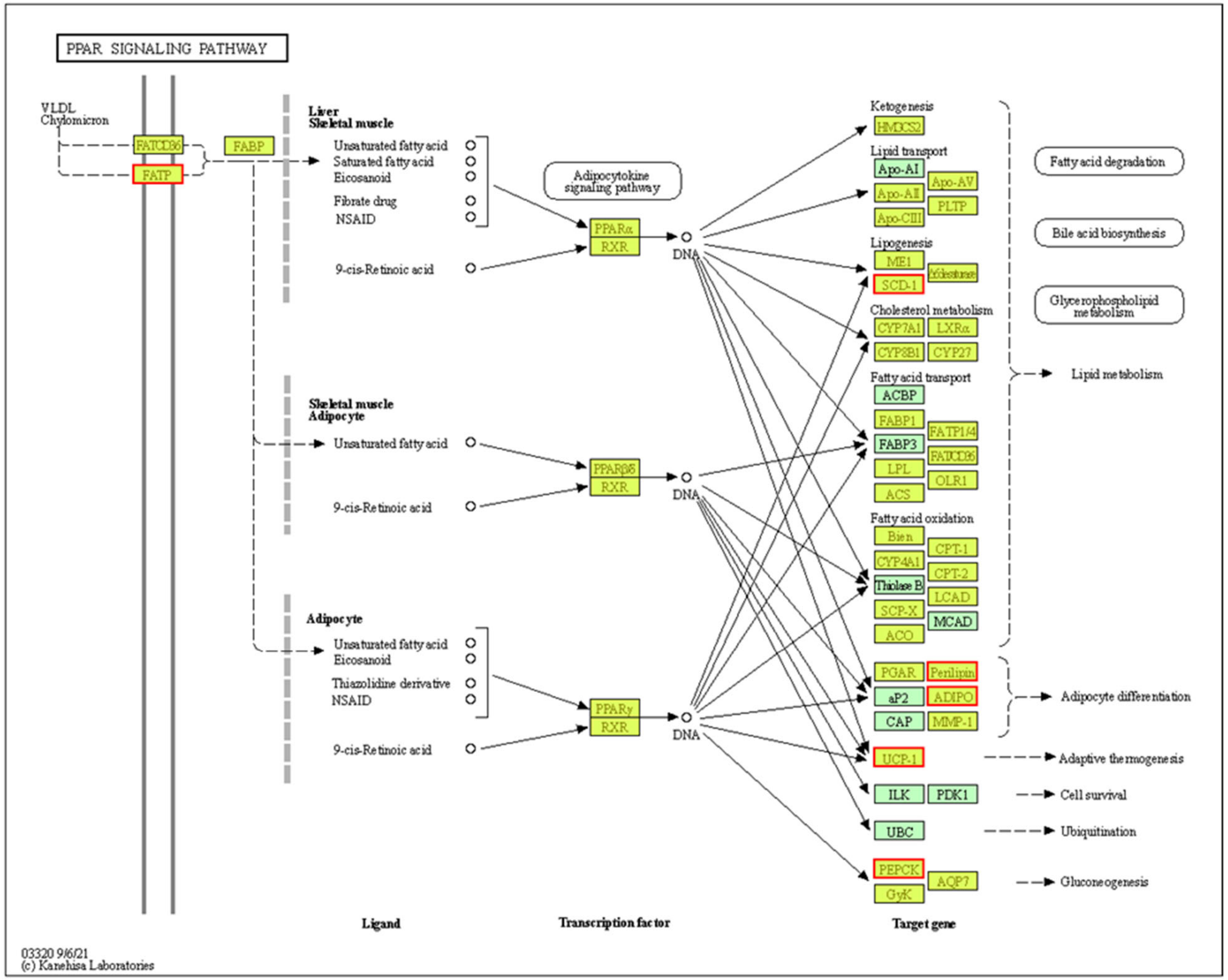


**Figure S4.** Enrichment of DEGs in PPAR signaling pathway.

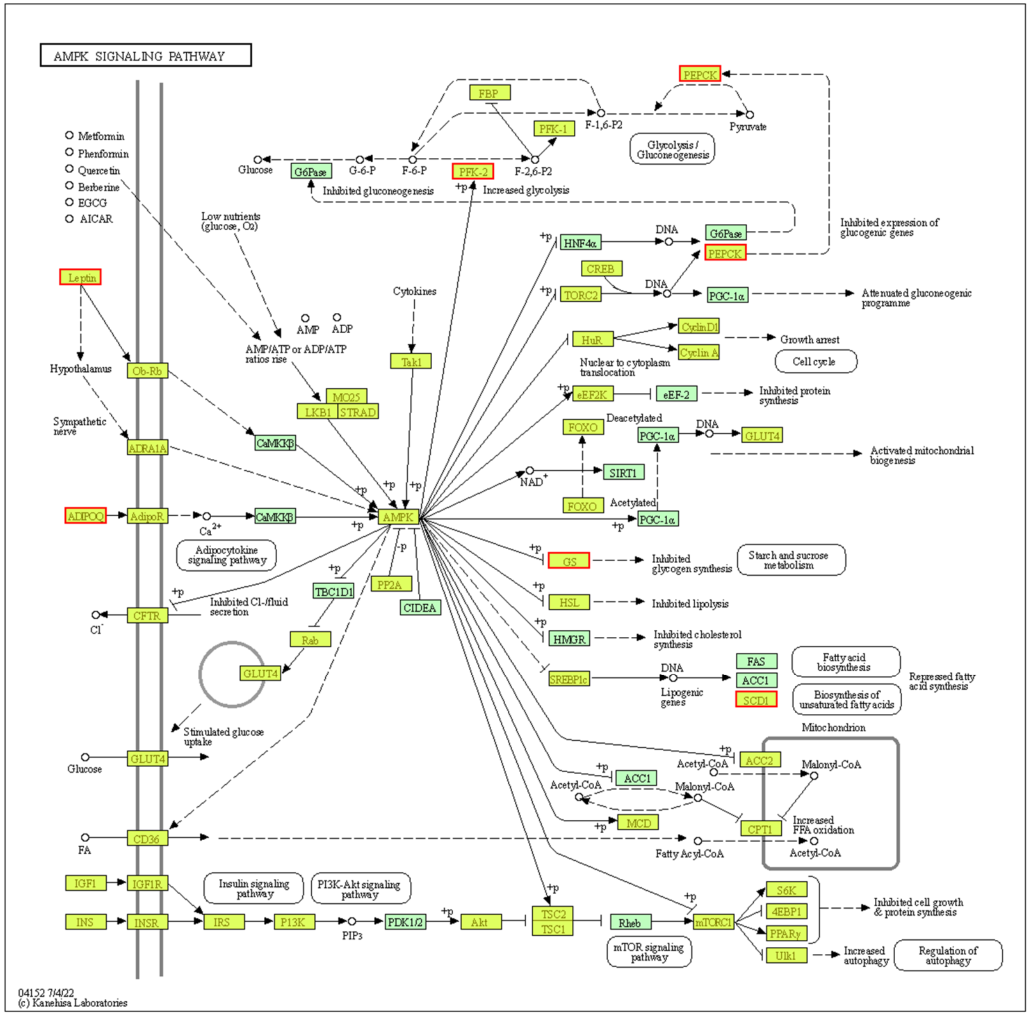


**Figure S5.** Enrichment of DEGs in AMPK signaling pathway.

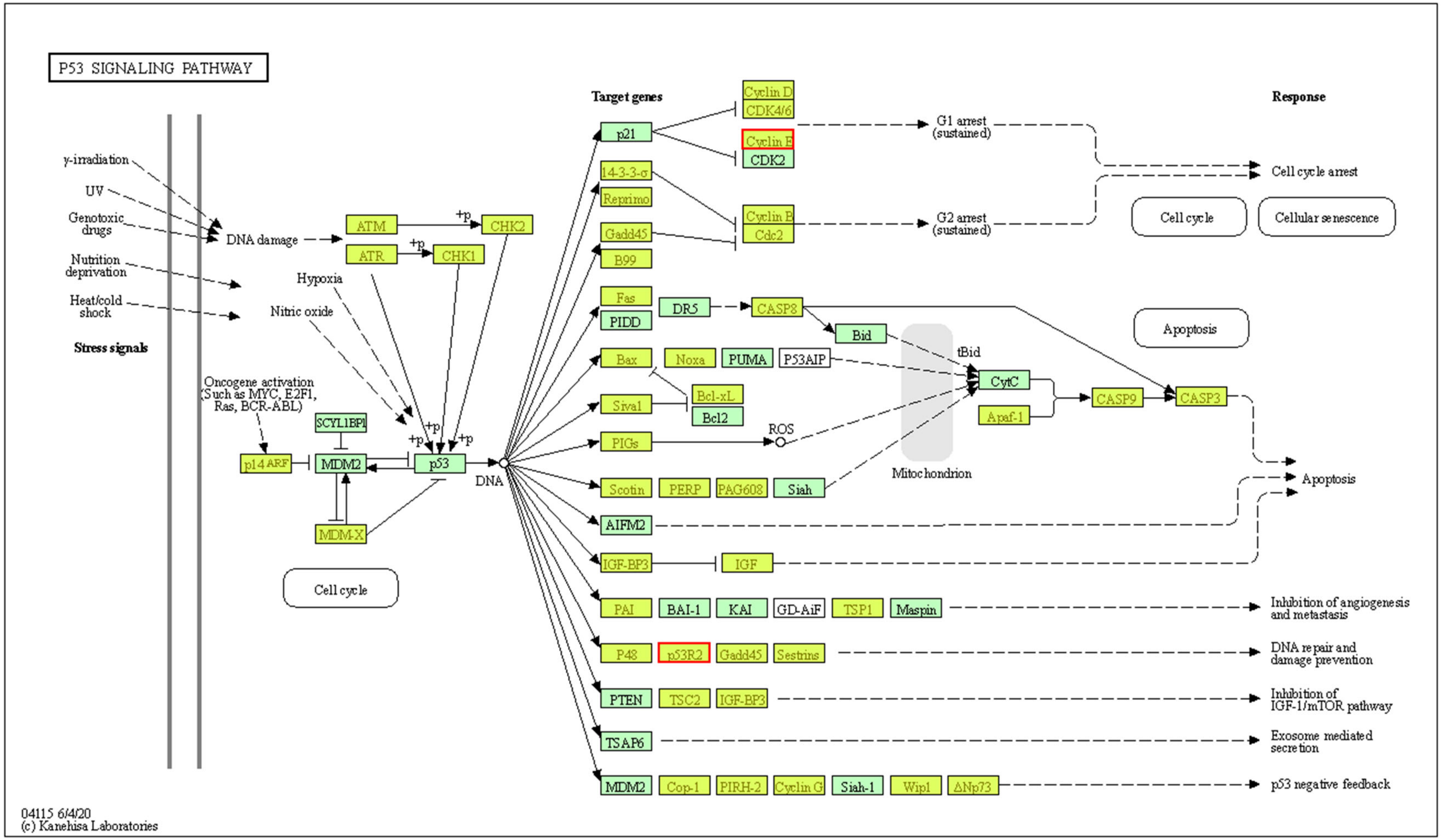


**Figure S6.** Enrichment of DEGs in P53 signaling pathway.

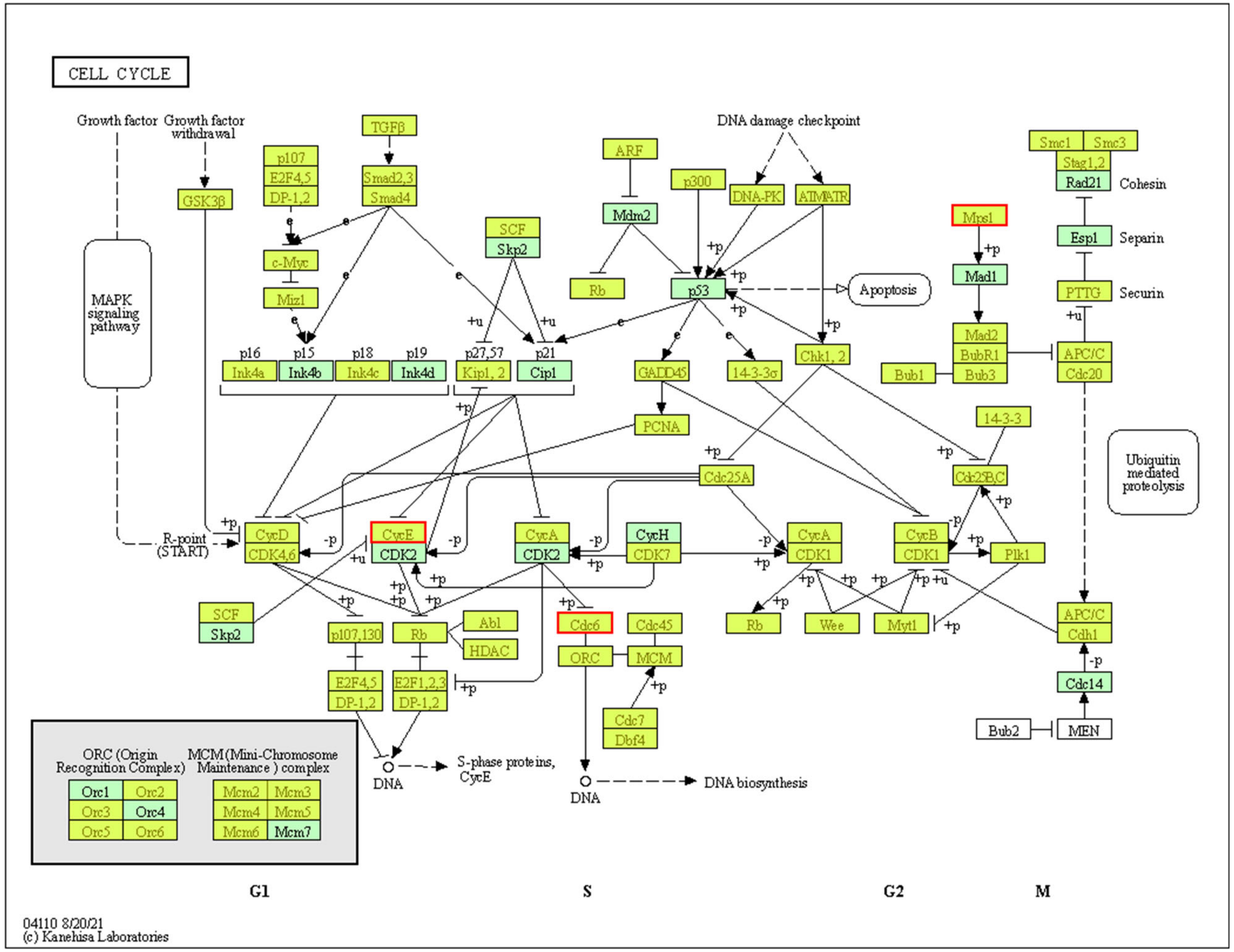


**Figure S7.** Enrichment of DEGs in cell cycle signaling pathway.

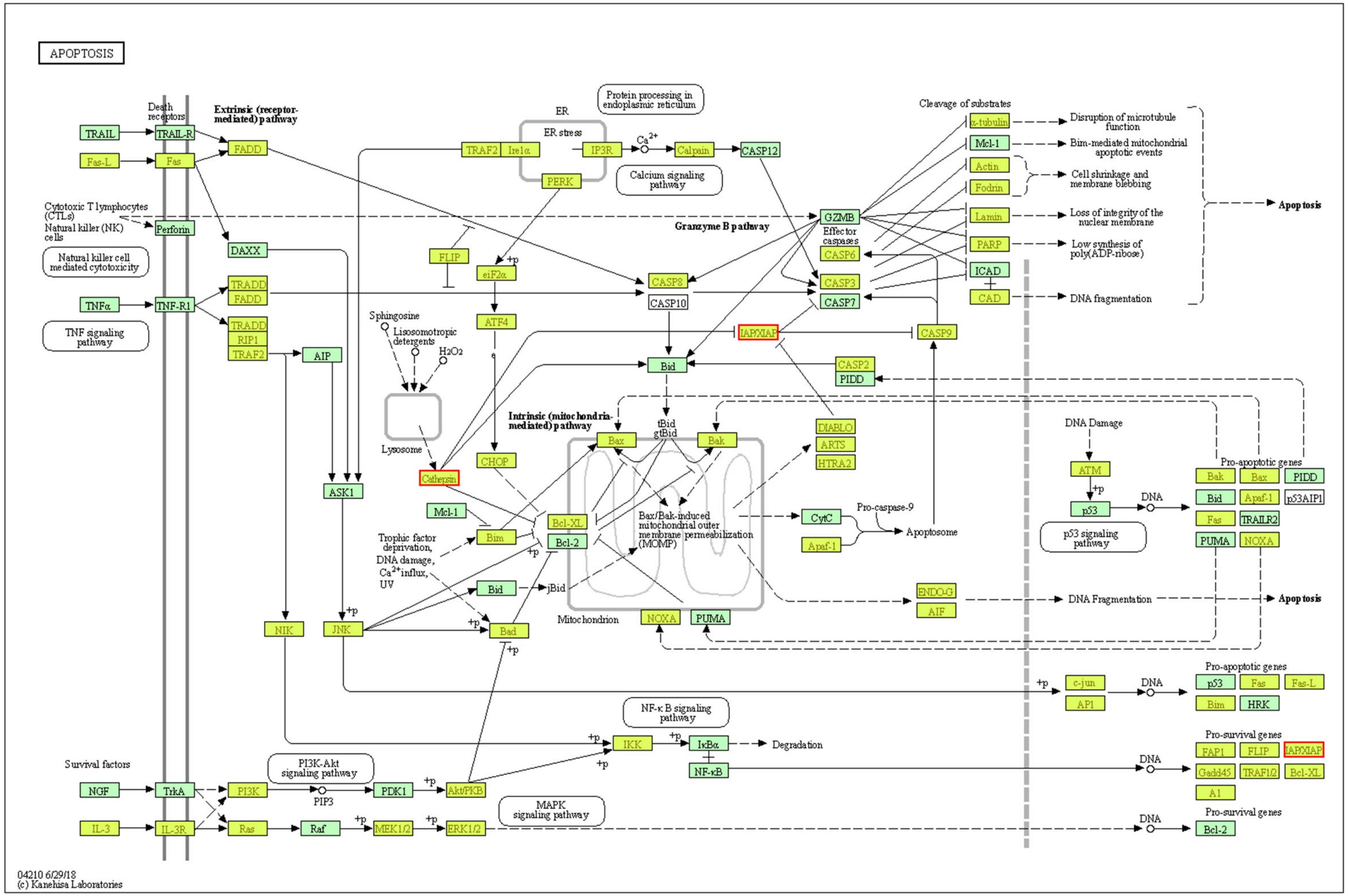


**Figure S8.** Enrichment of DEGs in apoptosis signaling pathway.

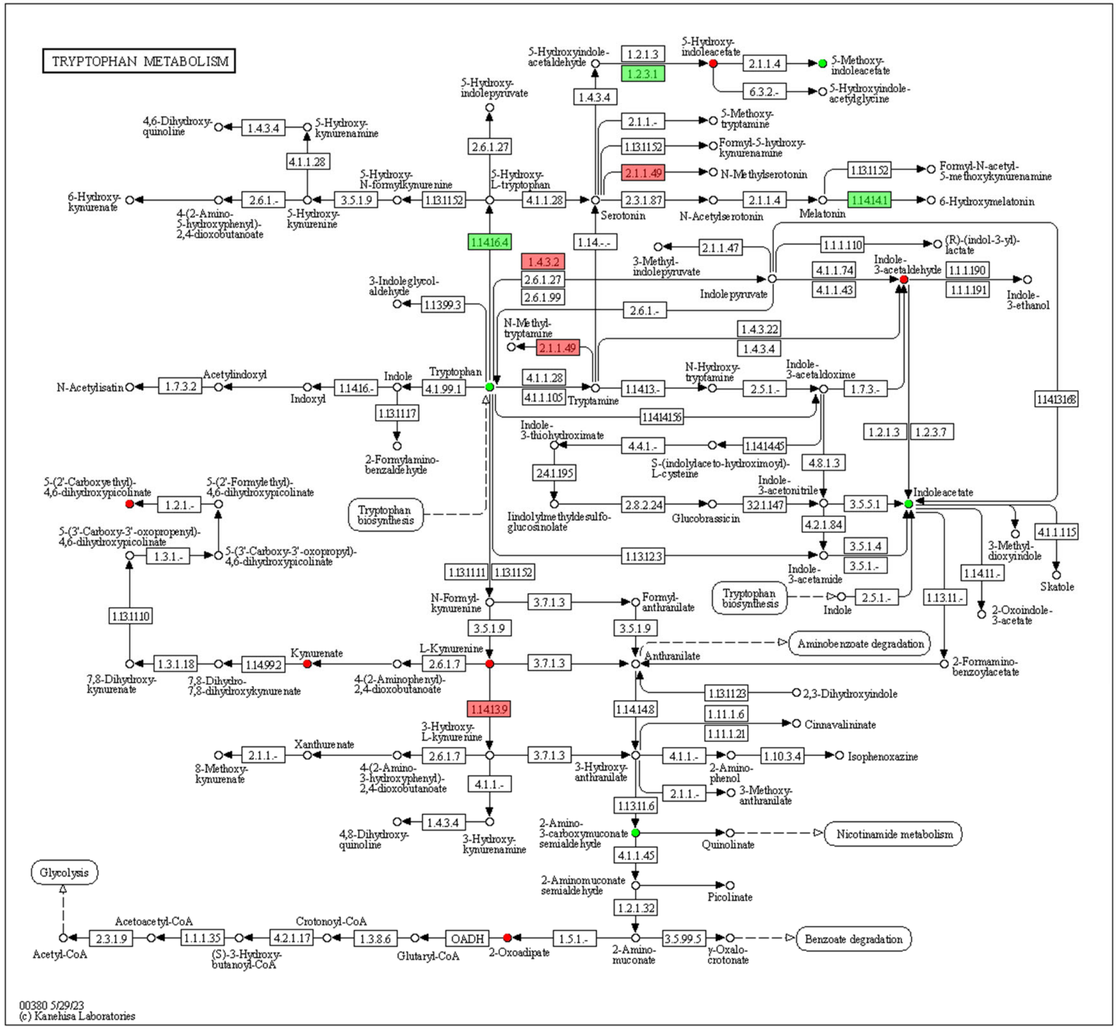


**Figure S9.** Enrichment of DEGs and DAMs in tryptophan metabolism pathway.

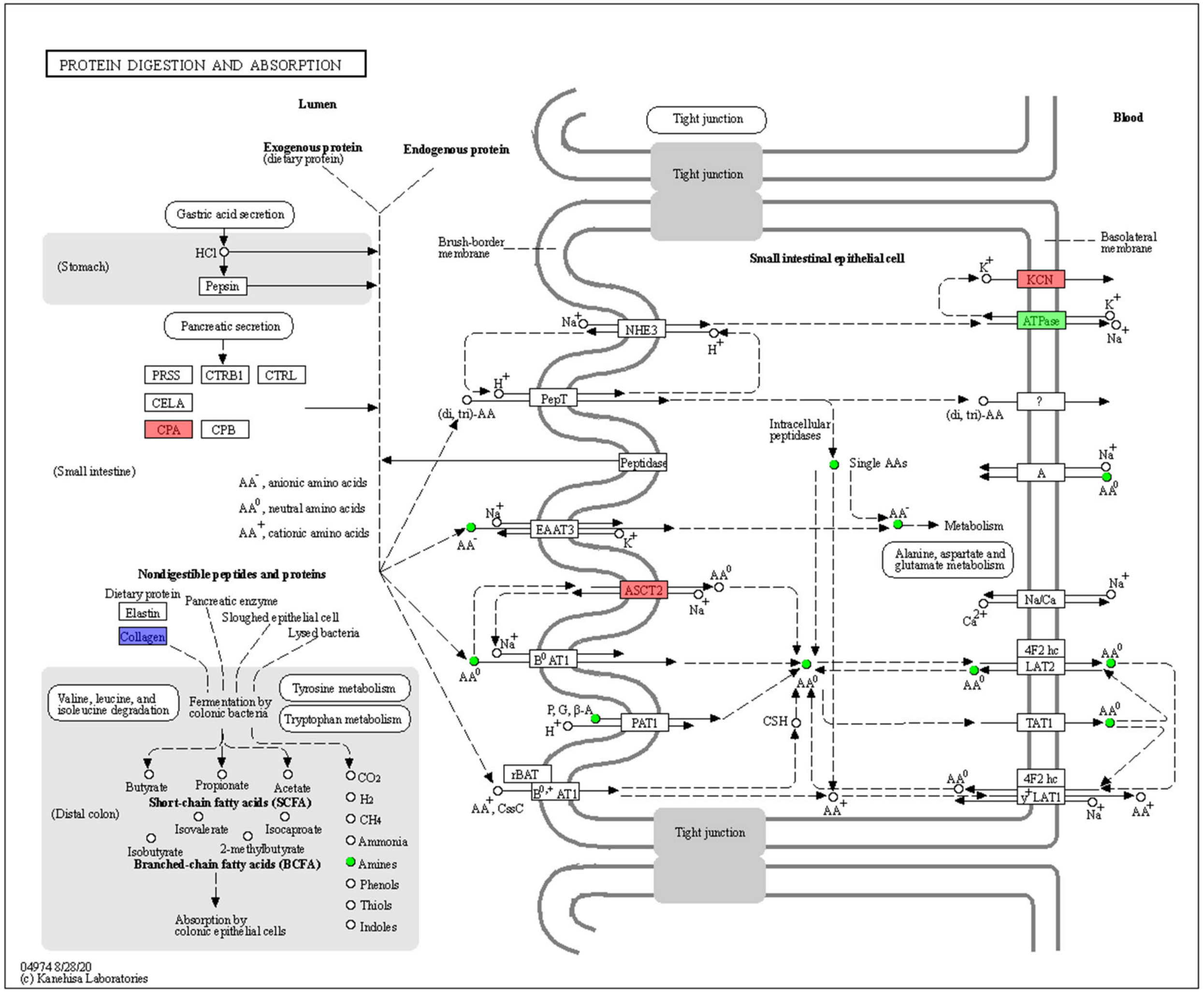


**Figure S10.** Enrichment of DEGs and DAMs in protein digestion and absorption pathway.
